# Wollastonite and Xonotlite from Industrial Waste as Functional Fillers in Self-Repairing Photocurable Cinnamon-Derived Vitrimer Composites

**DOI:** 10.1021/acsomega.6c03168

**Published:** 2026-09-23

**Authors:** Ramune Sidaraite, Tadas Dambrauskas, Sigita Grauzeliene, Jolita Ostrauskaite, Kestutis Baltakys

**Affiliations:** † Department of Silicate Technology, 70309 Kaunas University of Technology, Radvilenu Rd. 19, LT-50254 Kaunas, Lithuania; ‡ Department of Polymer Chemistry and Technology, Kaunas University of Technology, Radvilenu Rd. 19, LT-50254 Kaunas, Lithuania

## Abstract

This work aims to reuse calcium-rich waste from the sugar production industry as a precursor for the synthesis of calcium silicates and subsequently to apply the resulting calcium silicates as functional fillers in photocurable, catalyst-free vitrimer composites. To synthesize calcium silicate compounds, a mixture of calcium-rich waste and quartz, with a CaO/SiO_2_ molar ratio of 1.0, was prepared. Calcium silicates were synthesized using one-step solid-state calcination (400–1300 °C) and a two-step method combining hydrothermal treatment (200 °C, 16–72 h) with subsequent calcination (400–1300 °C). The one-step method led to the formation of multiple-phase mixtures, with wollastonite forming only above 1100 °C and coexisting with other phases. In contrast, the two-step synthesis enabled the formation of calcium silicate hydrates, tobermorite and Xonotlite, which recrystallized into β-wollastonite at 800–1000 °C and α-wollastonite at higher temperatures. Only the products obtained via the two-step route were selected as fillers due to their more suitable phase composition. The obtained Xonotlite and wollastonite were incorporated (10–20 wt %) into photocurable vitrimer matrices based on bisphenol A glycerolate dimethacrylate, 2-hydroxyethyl methacrylate, and cinnamyl methacrylate. Rheological analysis showed rapid photopolymerization for all formulations (gel point ≈1 s). Incorporation of cinnamyl methacrylate increased storage modulus and reduced polymerization shrinkage, while filler addition increased viscosity and stiffness but decreased storage modulus during curing. Self-welding at 180 °C confirmed dynamic bond exchange within the catalyst-free network. This work demonstrates an integrated circular approach in which industrial CaO waste is reused to synthesize functional calcium silicates that can be applied in vitrimer systems to enhance their properties.

## Graphical abstract

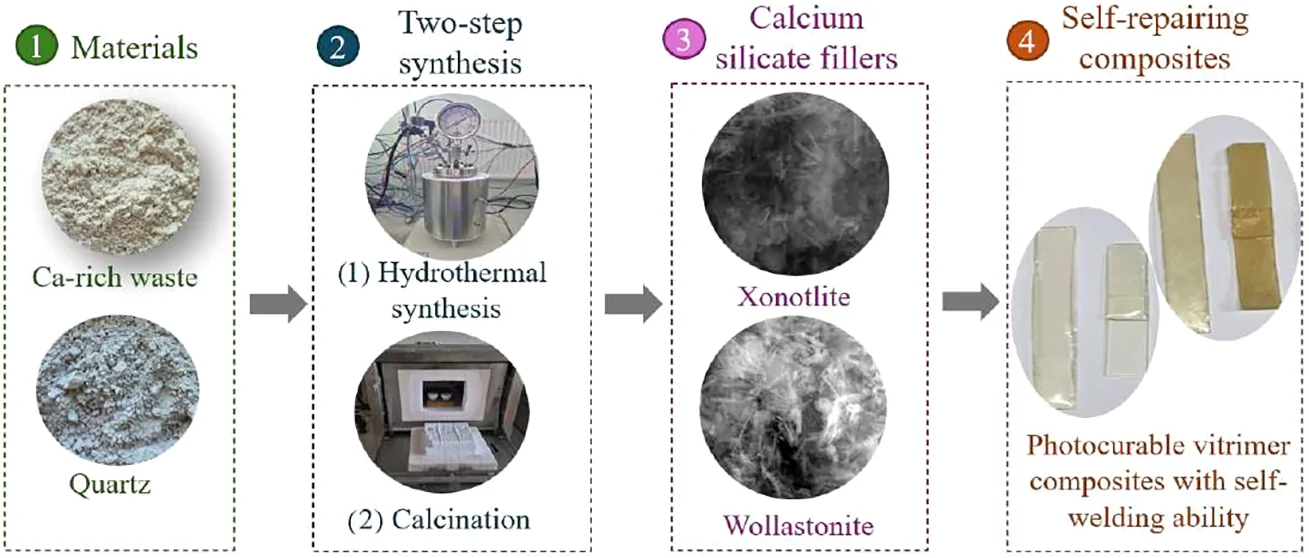

## 1 Introduction

Rising technological development, population growth, and intensified consumption are driving an increasing demand for nonrenewable resources while simultaneously generating enormous amounts of waste.^1–3^ In 2022, the European Union produced approximately 2.23 billion tons of waste, of which only 40.8% was recycled and 30.2% was landfilled.^4–6^ The recycling of industrial waste remains particularly challenging due to its complex, heterogeneous, and often contaminated composition.^7–9^ Nevertheless, such waste is a promising secondary resource that can partially replace natural raw materials, thereby contributing to circular economy objectives and reducing CO_2_ emissions.^10^

Calcium-rich industrial waste is especially attractive for the synthesis of calcium silicates and calcium silicate hydrates (CSH),^11,12^ offering an alternative to conventional ways that rely on the calcination of CaCO_3_.^13^ One significant but underexplored source is calcium-rich waste generated in the sugar production industry, with an estimated global annual production of 36.8 million tons.^14^ Despite its large availability, the scientific literature lacks systematic studies on the application of this waste for the synthesis of calcium silicates and CSH phases.

Calcium silicates, particularly wollastonite (CaSiO_3_), and CSH phases play an important role in environmentally oriented technologies, including low-carbon cement systems, adsorbents, catalyst supports, and biomedical materials.^15–19^ Wollastonite, characterized by a CaO/SiO_2_ molar ratio of 1.0, is widely applied in ceramics, construction materials, metallurgy, friction products, and as a functional filler in polymers and resins.^20–26^ Its properties and formation kinetics strongly depend on the composition and quality of the raw materials used for synthesis. While wollastonite has been successfully synthesized from various calcium-rich wastes such as eggshells, cement bypass dust, and ceramic sludge, no studies have addressed its production from calcium-rich waste originating from the sugar industry.^28–31^ Gineika et al.^27^ for the two-step synthesis of wollastonite used secondary raw material: AlF_3_ production byproduct—silica gel and laboratory-grade calcium oxide. The obtained results showed a high potential to use industrial wastes as raw materials; however, there is no data about the application of calcium-rich secondary raw material from the sugar production industry for wollastonite synthesis and application. The resulting phase composition, morphology, and physicochemical properties may significantly influence its potential industrial applications.

In parallel, vitrimer materials have emerged as a new class of permanently cross-linked polymer networks that combine the dimensional stability and insolubility of thermosets with the reprocessability of thermoplastics. This unique behavior is enabled by dynamic covalent bond exchange reactions that allow network topology rearrangement at elevated temperatures without depolymerization.^32^ This concept has attracted broad interest because vitrimer networks can be reshaped, repaired, and self-welded by heat while maintaining a cross-linked structure, making them particularly attractive for protective coating applications, where thermal repair and reprocessing can prolong coating lifetime, reduce maintenance requirements, and minimize material waste.^33^ Photocurable vitrimer systems are particularly attractive for coating applications due to solvent-free processing, rapid curing, and spatial control.^34^ Recent developments have focused on catalyst-free vitrimer concepts to avoid issues related to catalyst migration, long-term stability, and processing constraints while maintaining sufficient bond exchange rates at elevated temperatures.^35–37^

For coating applications, the incorporation of inorganic fillers is a common strategy to enhance stiffness and mechanical performance.^38^ However, fillers may also restrict polymer chain mobility and dynamic bond exchange, potentially affecting self-welding efficiency and network rearrangement. This creates a clear need to investigate vitrimer composites containing mineral fillers such as calcium silicates, where filler morphology and particle–matrix interactions may influence curing behavior, mechanical performance, and thermally activated self-welding capability.

The present study proposes an integrated circular approach: calcium-rich waste from the sugar industry is reused as a precursor for the synthesis of valuable calcium silicate phases (wollastonite and Xonotlite), which are subsequently employed as functional fillers in photocurable, catalyst-free vitrimer composites for coating applications. To the best of our knowledge, such a combination—synthesizing calcium silicates from sugar industry CaO waste and incorporating them into dynamic covalent vitrimer matrices—has not been previously reported. The resin formulation was designed using bisphenol A glycerolate dimethacrylate (BisGMA) (Figure 1) as a rigid, bifunctional methacrylate monomer providing high cross-linking density and mechanical strength.^39^ 2-Hydroxyethyl methacrylate (HEMA) and cinnamyl methacrylate (CMA) can both be derived from renewable resources and were incorporated to introduce hydroxyl and ester functional groups into the network, enabling dynamic bond exchange while contributing to network rigidity. The presence of hydroxyl groups is essential for facilitating transesterification reactions in catalyst-free vitrimer systems. HEMA can be derived from intermediates obtained from bioethanol,^40^ whereas CMA can be synthesized from cinnamyl alcohol, which originates from naturally occurring cinnamic acid derivatives present in cinnamon oil and lignocellulosic biomass.^41^ Ethyl­(2,4,6-trimethylbenzoyl) phenylphosphinate (TPOL) was selected as an efficient photoinitiator suitable for UV/vis curing, ensuring rapid polymerization and high conversion without the need for additional catalysts.^42^ While vitrimer composites have been widely reported, most studies focus on thermally cured networks with catalysts.^43^ In contrast, this work presents a photocurable, catalyst-free vitrimer composite system without external catalysts, incorporating calcium silicate fillers (wollastonite and Xonotlite) that have not yet been used in dynamic covalent matrices. Accordingly, the objectives of this work were: (I) to evaluate one-step and two-step routes for producing wollastonite/Xonotlite from sugar-industry calcium-rich waste, (II) to characterize the phase composition, morphology, density, surface area, and particle size distribution of the synthesized products, and (III) to assess their suitability as functional fillers in photocurable, catalyst-free vitrimer composites intended for repairable coating applications.

**Figure 1:**
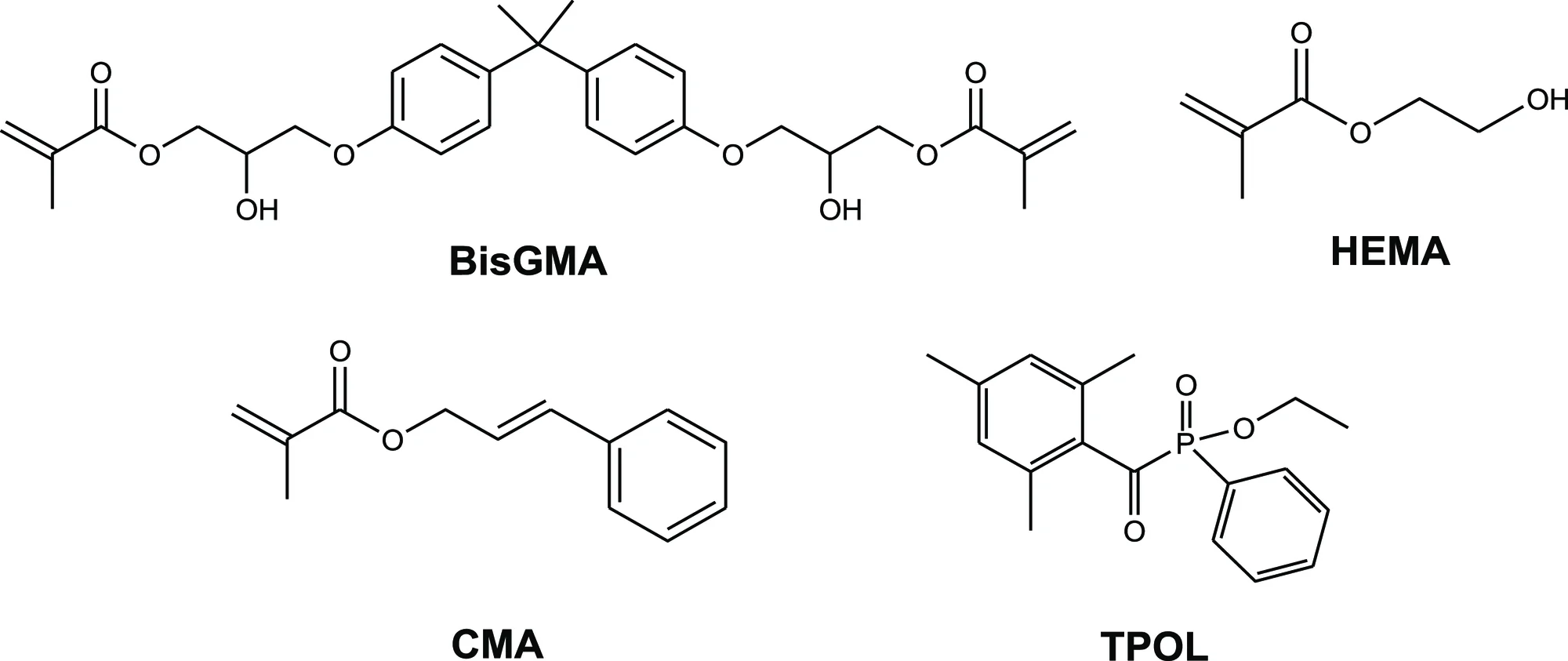
Chemical structures of bisphenol A glycerolate dimethacrylate (BisGMA), 2-hydroxyethyl methacrylate (HEMA), cinnamyl methacrylate (CMA), and ethyl­(2,4,6-trimethylbenzoyl) phenylphosphinate (TPOL).

## 2 Methodology

### 2.1 Materials

In this work, the following raw materials were used:

(1) Calcium-rich waste from the sugar production industry (labeled as SWC) was used as a calcium source for the synthesis of calcium silicates. The mineralogical composition of the initial SWC was characterized by X-ray diffraction (XRD) (Figure 2). The XRD pattern showed that portlandite (Ca­(OH)_2_) and calcium carbonate (CaCO_3_) were the main crystalline phases together with minor amounts of other mineral phases associated with impurities present in the industrial waste. Prior to use, SWC was milled for 7.5 min at 750 rpm using a vibrating mill and subsequently calcined at 900 °C for 1 h in a high-temperature furnace (Snol 8.2 1100, Lithuania). The mass loss during calcination at 1000 °C was 30.4 wt %, indicating the removal of volatile components, mainly CO_2_ from carbonate decomposition and structurally bound water. The chemical composition was determined for the calcined material, since this was the reactive precursor used in the synthesis. The free CaO content after calcination was 91.8 wt %, and the oxide composition of the calcined SWC is presented in Table 1.

(2) Quartz sand (SiO_2_, “Anyksciu kvarcas’’, Lithuania), which was milled for 35 min at 950 rpm using a vibrating mill (Fritsch Pulverisette 9, Germany). The SiO_2_ content is 98.5 wt %. The mineralogical composition of the quartz sand was additionally characterized using X-ray diffraction analysis (XRD), which showed that it is quartz, also known as silicon dioxide (SiO_2_) (Figure 3).

(3) Bisphenol A glycerolate dimethacrylate (BisGMA), 2-hydroxyethyl methacrylate (HEMA), and ethyl­(2,4,6-trimethylbenzoyl) phenylphosphinate (TPOL) were purchased from Merck. Cinnamyl methacrylate (CMA) was purchased from Specific Polymers. Silica dimethylsilylate (AEROSIL R972) was purchased from Evonik. All of the materials were used as received (Table 1).

**Table 1:** Oxide Composition of SWC Determined by XRF

oxide	CaO	SiO_2_	K_2_O	MgO	Al_2_O_3_	SO_3_	Fe_2_O_3_	Na_2_O	other
concentration, wt %	91.8	1.71	1.29	1.14	0.67	0.55	0.41	0.14	2.29

**Figure 2:**
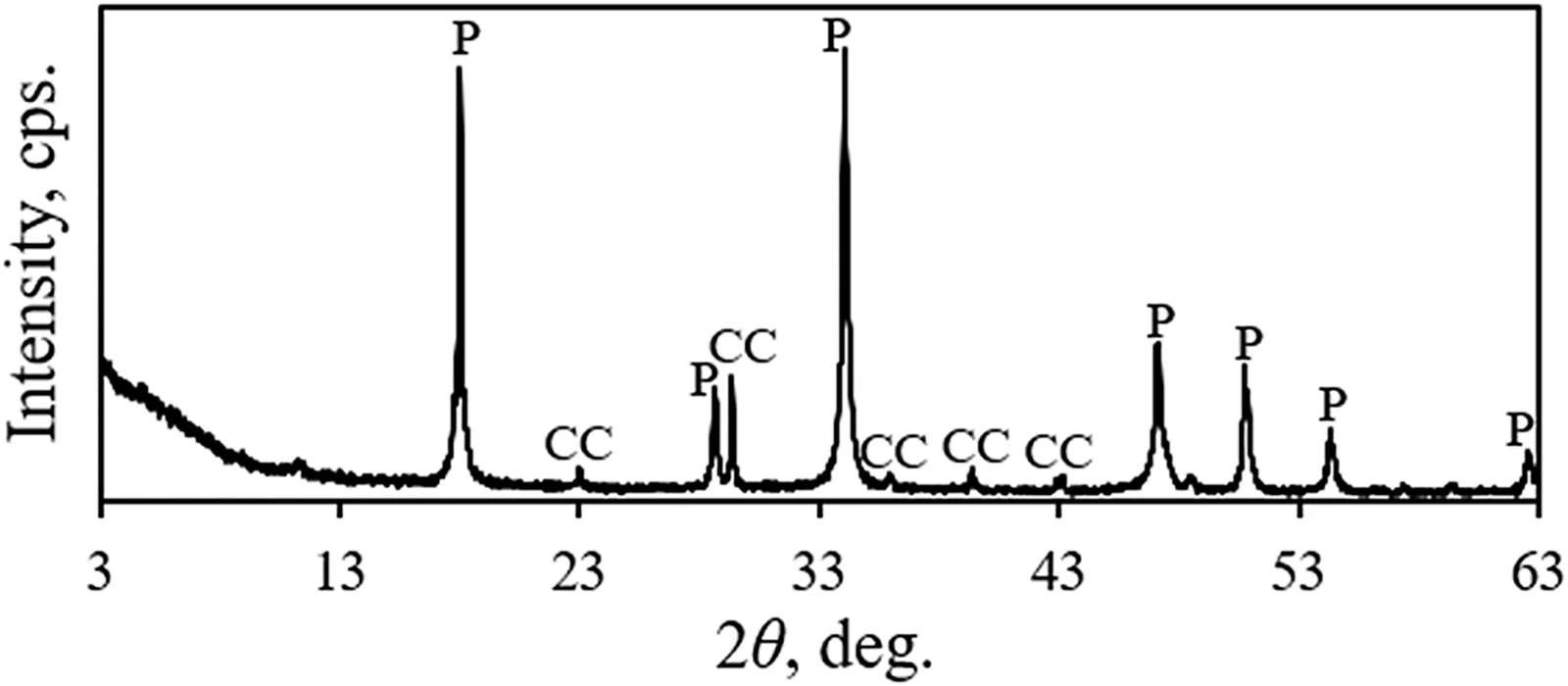
XRD curve of SWC sample. Indices: P—portlandite, CC—calcium carbonate.

**Figure 3:**
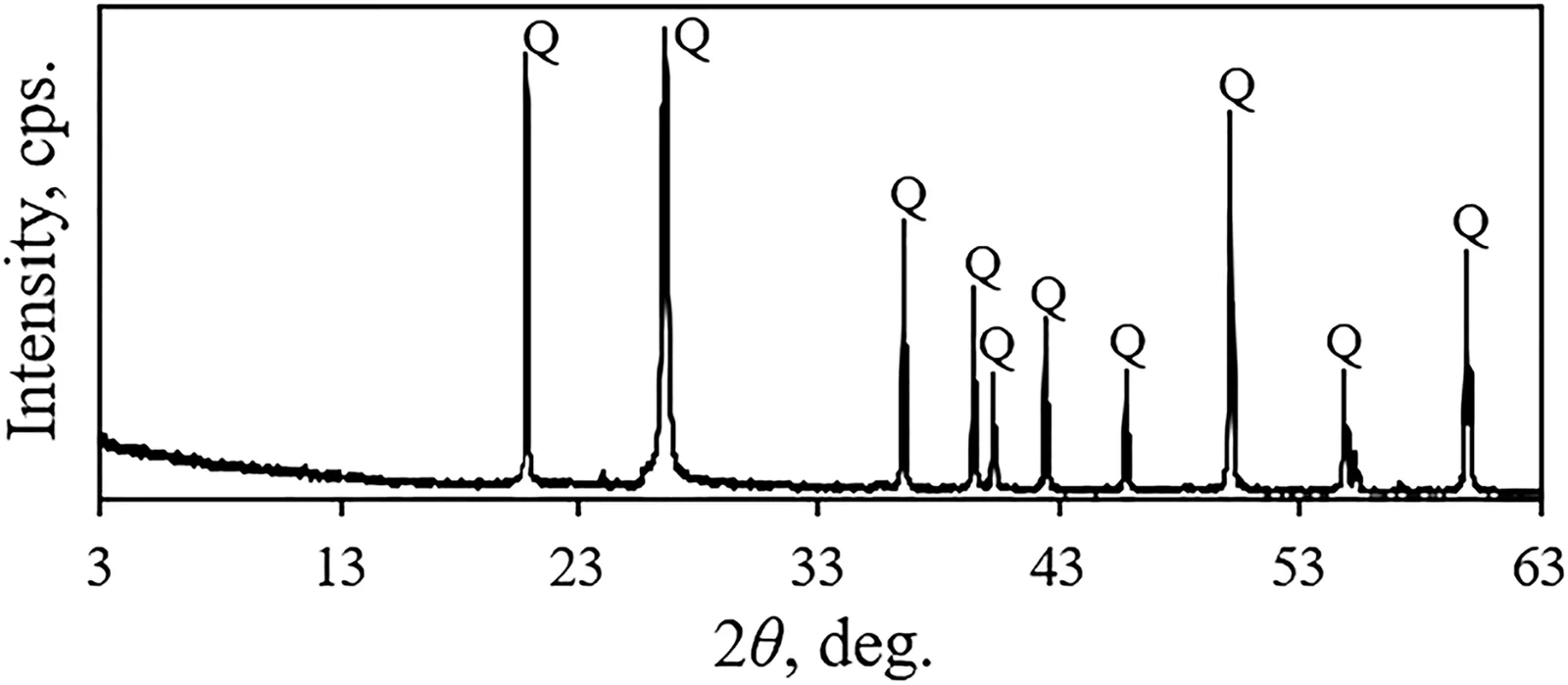
XRD curve of quartz sand sample. Index: Q—quartz.

### 2.2 Synthesis Methods

For hydrothermal synthesis, calcium oxide obtained from sugar industry waste was mixed with milled quartz sand at a CaO/SiO_2_ molar ratio of 1.0. The powders were homogenized for 1 h using a Turbula Type T2F mixer (Switzerland) to ensure uniform distribution. 2 g of the homogenized dry mixture was placed in 25 mL polytetrafluoroethylene-lined vessels and subsequently dispersed in distilled water to achieve a water-to-solid mass ratio of 10:1.

Hydrothermal synthesis was conducted in unstirred suspensions using vessels placed inside a stainless-steel autoclave (Parr Instruments, IL). The autoclave was heated to 200 °C under saturated steam pressure, reaching the target temperature within 2 h. Isothermal treatment was maintained for durations ranging from 0 to 72 h. Upon synthesis, the autoclave was left for 3 h to cool down to room temperature. The resulting suspensions were filtered, washed with acetone to minimize carbonation, dried at 80 ± 5 °C for 24 h, and sieved to obtain powders with particle sizes below 80 μm.

Wollastonite was synthesized via two different methodologies. In the one-step method, the dry SWC–quartz mixture (CaO/SiO_2_ = 1.0) was directly calcined in a chamber furnace (Nabertherm B 180, Germany) at temperatures between 400 and 1300 °C for 1 h, using a heating rate of 500 °C/h.

In the two-step method, hydrothermally synthesized products obtained after 16 and 72 h at 200 °C were used as precursors and subjected to calcination under identical furnace conditions (Figure 4, 400–1300 °C, 1 h, heating rate 500 °C/h).

**Figure 4:**
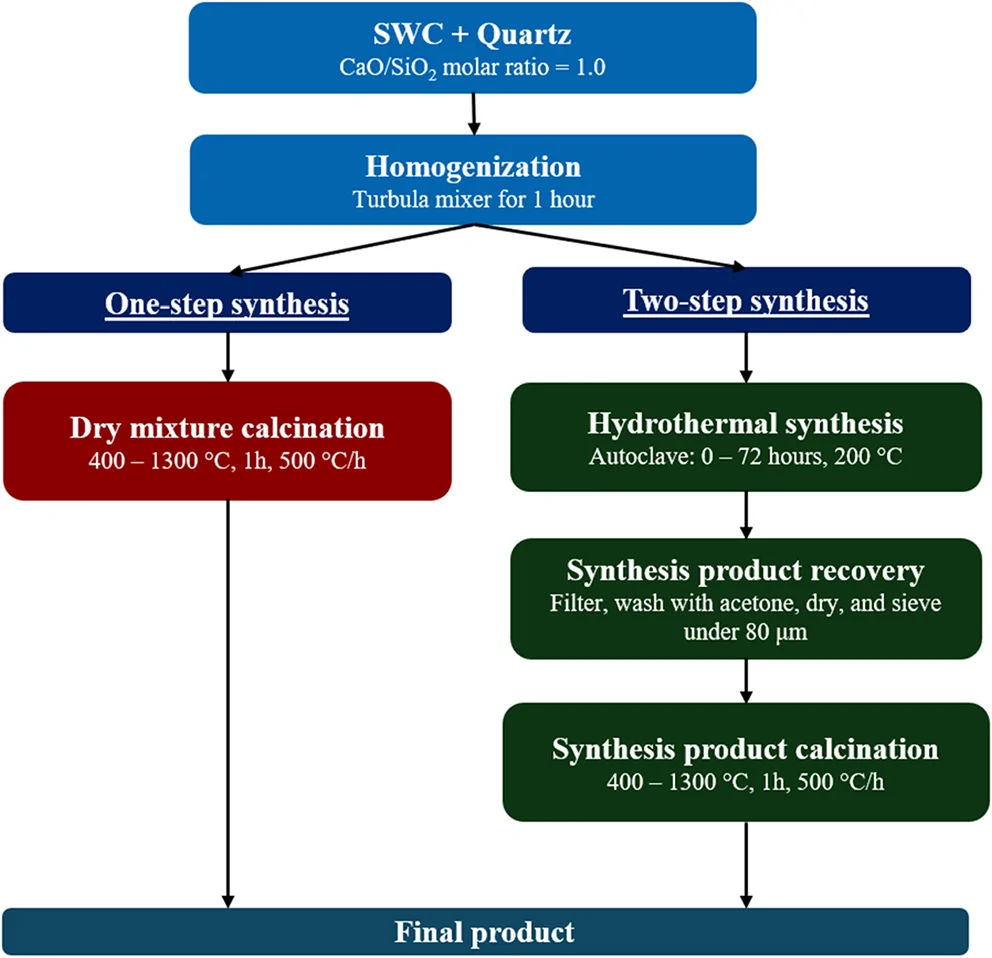
Schematic illustration of the one-step and two-step synthesis routes used for wollastonite preparation from SWC and quartz sand.

### 2.3 Composition of Photocurable Resins and Cross-Linking Process

Initial resin mixtures were prepared by combining 50 wt % of BisGMA with either 50 wt % of HEMA or a mixture of 25 wt % HEMA and 25 wt % of CMA. 3 mol % of TPOL was added as a photoinitiator. The components were mixed at 22 °C for 20 min under mechanical stirring until a homogeneous formulation was obtained.

For composite formulations, wollastonite or Xonotlite was incorporated at 10 or 20 wt % into the corresponding resin mixture and dispersed using mechanical stirring until a uniform particle distribution was achieved. 0.1 wt % of Aerosil R972 was added as an antisettling agent. The letters B, H, C, W, and X correspond to BisGMA, HEMA, CMA, wollastonite, and Xonotlite, respectively, and the numbers indicate weight percentages in the formulation (Table 2 and Figure 5).

**Table 2:** Composition of the Resins and Composite Preparation

sample	monomer ratio BisGMA:HEMA:CMA (wt %)	filler	amount of filler (wt %)	viscosity (mPa·s)
**50B/50H**	50:50:0		0	111.23 ± 0.62
**50B/25H/25C**	50:25:25		0	125.30 ± 1.86
**10W**	50:25:25	wollastonite	10	321.99 ± 18.07
**20W**	50:25:25	wollastonite	20	1541.45 ± 94.15
**10X**	50:25:25	Xonotlite	10	316.77 ± 3.50
**20X**	50:25:25	Xonotlite	20	1129.10 ± 50.70

**Figure 5:**
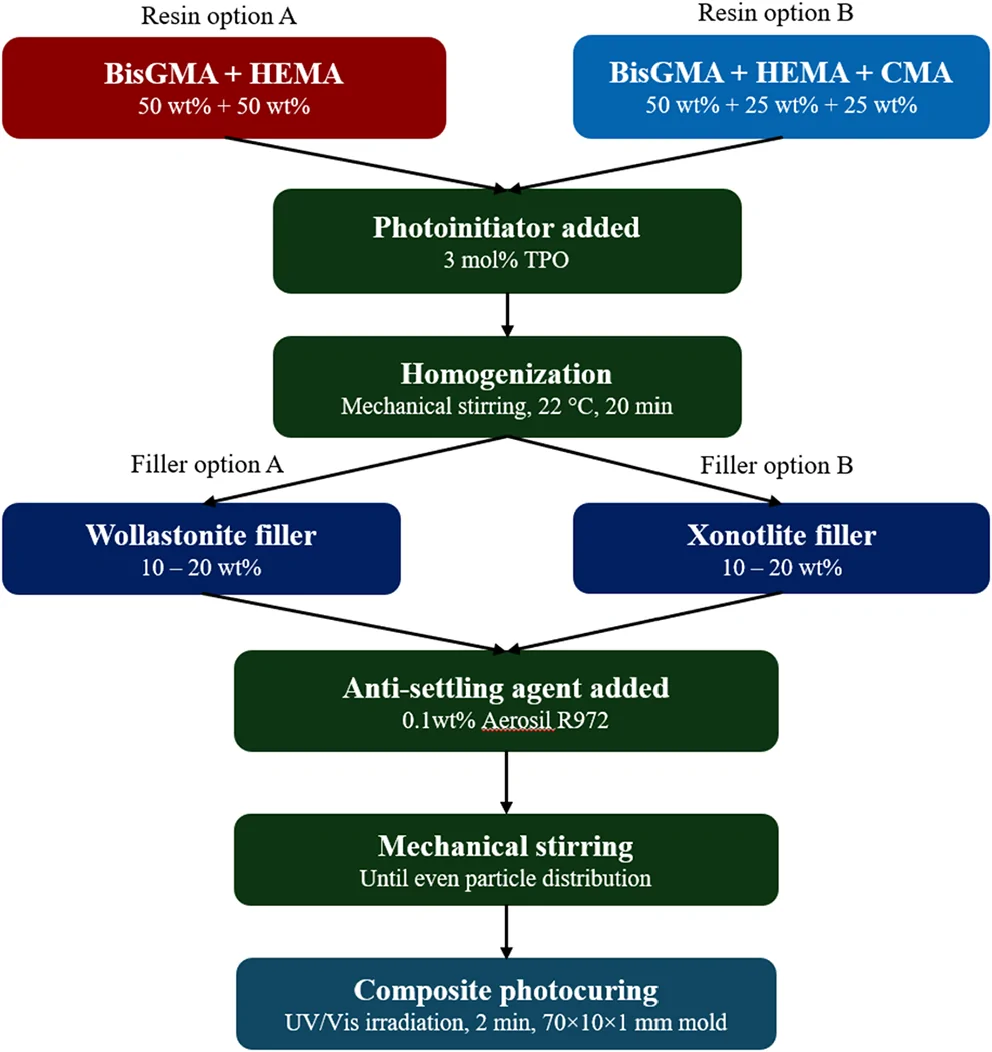
Schematic illustration of photocurable vitrimer resin and composite preparation.

Samples were prepared by photocuring the resin in a Teflon mold (70 × 10 × 1 ± 0.01 mm) under UV/vis irradiation using a 500 W Helios Italquartz GR.E lamp (250–450 nm, 310 mW·cm^–2^) for 2 min.

### 2.4 Characterization Methods

#### 2.4.1 Photocuring and Rheological Properties of the Resins

The kinetics of photocuring and the rheological properties of the resins were investigated using an Anton Paar MCR302 rheometer equipped with a plate–plate geometry and an OmniCure S2000 irradiation device (Lumen Dynamics Group Inc.). Resin viscosity was measured over a shear rate range of 0.001 to 50 s^–1^ at 25 °C for a duration of 60 s. Photocuring tests were performed in shear mode with three repetitions, at a frequency of 10 Hz, for a total duration of 300 s. UV/vis irradiation was initiated 30 s after the start of the test. During the experiment, the storage modulus (*G*′), loss modulus (*G*″), complex viscosity, layer shrinkage, and gel point were recorded. The gel point (*t*_gel_) was determined as the crossover point of the *G*′ and *G*″ curves.

#### 2.4.2 Instrumental Analysis of Raw Materials and Synthesis Products

The chemical structure of the polymers was verified by Fourier transform infrared (FT-IR) spectroscopy. FT-IR spectra were recorded using a Spectrum BX II FT-IR spectrometer. The spectra were recorded in reflection mode over the wavenumber range of 650–4000 cm^–1^.

Phase composition of synthesis products was determined using X-ray diffraction (XRD) powder analysis on a D8 Advance diffractometer (Bruker AXS, Karlsruhe, Germany) operating at a tube voltage of 40 kV and tube current of 40 mA. The X-ray beam was filtered with a Ni 0.02 mm filter to select the Cu Kα wavelength. Diffraction patterns were recorded in a Bragg–Brentano geometry using a fast-counting detector, Bruker LynxEye (Bruker AXS, Karlsruhe, Germany), based on silicon strip technology. Specimens were scanned over a range of 3–70° (2θ) at a scanning speed of 6°/min using a coupled two theta/theta scan type.

The simultaneous thermal analysis was conducted using a Linseis PT1000 instrument (Linseis Massgeraete GmbH, Selb, Germany) under the following conditions: heating rate of 15 °C/min, temperature range 30–1000 °C, nitrogen atmosphere, ceramic sample handlers, platinum crucibles, and sample mass of approximately 13 mg.

The amount of free CaO was determined according to standard ASTM C114–11b.

The materials’ particle size distribution and specific surface area were analyzed using a CILAS 1090 LD particle analyzer, capable of measuring within the 0.04–500 μm range.

An FEI Helios Nanolab 650 scanning electron microscope (SEM) equipped with an energy-dispersive X-ray spectrometer (Oxford Instruments, High Wycombe, UK, INCA 4.15 software) was used for the surface investigation of the samples.

## 3 Results and Discussion

### 3.1 One-Step Wollastonite Synthesis

In the first step of this work, the possibility of using SWC for the one-step synthesis of wollastonite was investigated.^12^ For this reason, a dry mixture of SWC and quartz sand with a C/S ratio equal to 1.0 was prepared and later calcined at temperatures ranging from 400 to 1300 °C for 1 h.

It was determined that, within the temperature range of 400–700 °C, the initial mixture remained stable, as no new compounds formed and no significant changes in the diffraction peak area for either quartz {SiO_2_ (PDF 00-046-1405)} or calcium oxide {CaO (PDF 01-080-7710)} were observed (Figures 6a and 7). At a higher temperature (800 °C), a significant reduction in the area of the main diffraction peak of calcium oxide (from 12.1 to 9.2 cps) was observed. Meanwhile, the area of the main diffraction peak of quartz decreased slightly. This can be explained by the beginning of larnite {Ca_2_SiO_4_ (PDF 00-033-0302)} formation, which requires two times more calcium oxide than silicon dioxide. The formation of a higher-basicity compound (C/S = 2.0) than that of the initial mixture (C/S = 1.0) can be explained by the higher reactivity of calcium oxide. It was determined that calcium oxide fully reacted at 1000 °C, while traces of quartz were still present in the products even after calcination at 1300 °C (Figure 6a).

**Figure 6:**
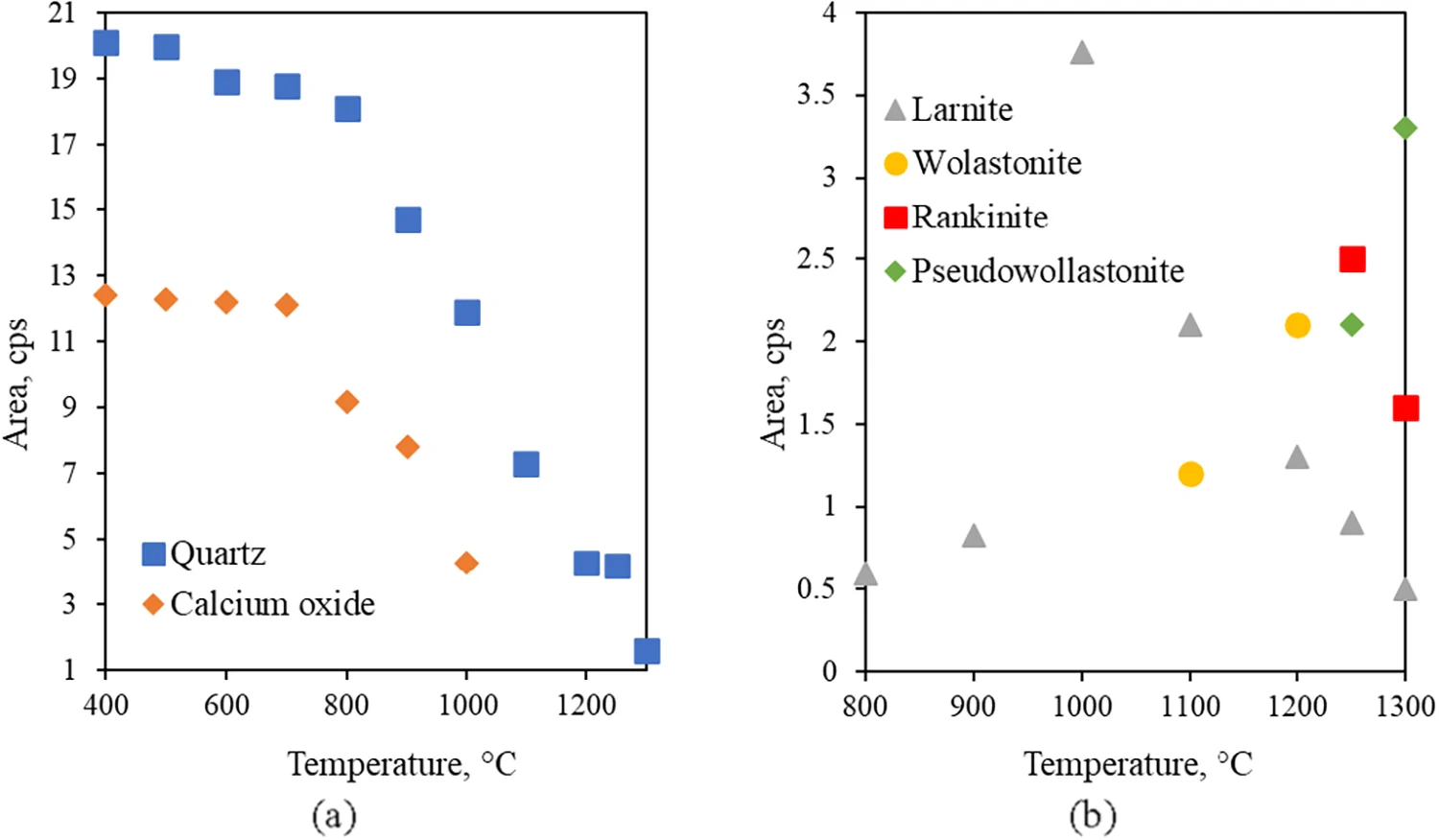
(a) Raw material diffraction peak area intensity dependency on the calcination temperature. (b) Product diffraction peak area intensity dependency on the calcination temperature.

**Figure 7:**
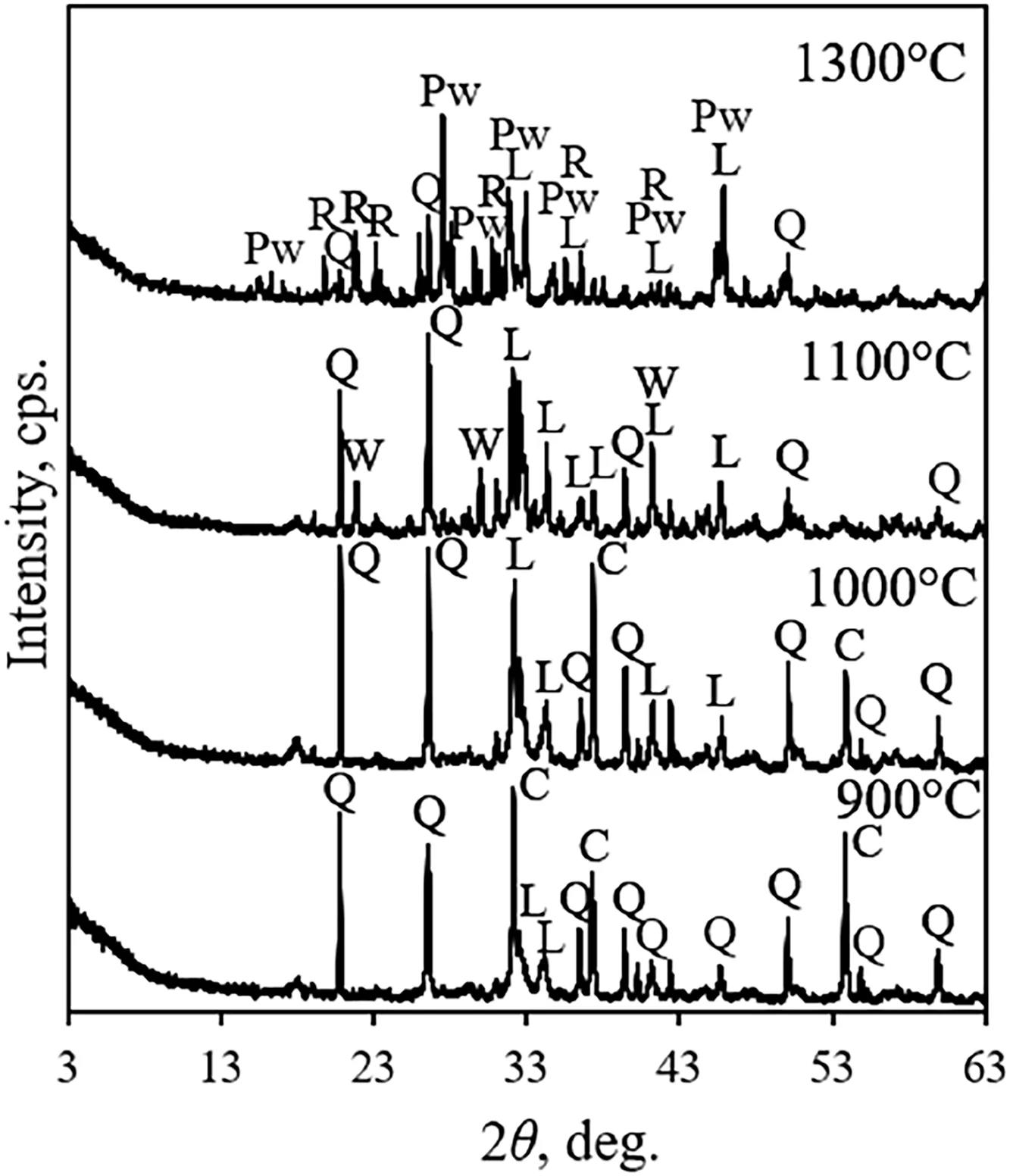
XRD curves of calcinated raw material. Indices: Q—quartz, C—calcium oxide, L—larnite, W—wollastonite, R—rankinite, Pw—pseudowollastonite.

The increase in calcination temperature from 800 to 1000 °C positively affected the formation of larnite because the area of its main diffraction peak increased from 0.6 to 3.8 (Figure 6b). A further increase in temperature (to 1300 °C) led to the recrystallization of larnite to other silicates, which was confirmed by a consistent decrease in the diffraction peak area to 0.5.

The target synthesis product (wollastonite) formed at 1100 °C (Figures 6a and 7). These data coincide with the data reported in the scientific literature.^27,44^ It was determined that β-wollastonite {β–CaSiO_3_ (PDF 04-016-5569)} formed in the temperature range of 1100–1200 °C. At 1250 °C, the latter phase became metastable and recrystallized into pseudowollastonite {α-CaSiO_3_ (PDF 01-080-9543)}. However, rankinite {Ca_3_Si_2_O_7_ (PDF 00-023-0214)} also formed together with pseudowollastonite. It was determined that an increase in calcination temperature to 1300 °C positively affected the formation of pseudowollastonite, as the area of its main diffraction peak (*d*-spacing = 0.275 nm) increased from 2.1 (1250 °C) to 3.3 (1300 °C), while that of rankinite decreased from 2.6 (1250 °C) to 1.6 (1300 °C).

The results showed that calcination of the SWC and quartz mixture, as a one-step wollastonite production process, was not successful because of the formation of various other calcium silicates, such as larnite and rankinite. This can be attributed to the low reactivity of quartz and the higher reactivity of calcium oxide, which led to the formation of higher-basicity calcium silicates at lower temperatures.

The results showed that calcination of the SWC and quartz mixture as a one-step wollastonite production process was not successful due to the formation of other calcium silicates, such as larnite and rankinite. This can be attributed to the low reactivity of quartz and the higher reactivity of calcium oxide, which promoted the formation of higher-basicity calcium silicates at lower temperatures. Wollastonite was formed only at 1100 °C, while pseudowollastonite, unreacted quartz, and other calcium silicate phases were still observed at higher temperatures. These results indicated that the direct solid-state reaction between the calcium and silica components was incomplete. Therefore, a two-step synthesis route involving hydrothermal treatment followed by calcination was investigated.

### 3.2 Two-Step Wollastonite Synthesis

A two-step synthesis combining the hydrothermal synthesis of precursors (usually calcium silicate hydrates) and their subsequent calcination can be used for the synthesis of wollastonite. Precursors can be synthesized over a wide temperature (130–220 °C) and duration (0–72 h) range. The most favorable temperature is around 200 °C. The molar ratio of the initial mixture should be around 1.0. The obtained precursors should be calcined at 800–1200 °C to form wollastonite.^27,45–47^

For this reason, in the next part of this work, the possibility of using SWC for the two-step synthesis of wollastonite was evaluated. The hydrothermal synthesis of precursors was conducted at 200 °C for 0–72 h.

#### 3.2.1 Hydrothermal Synthesis of Precursors (Xonotlite)

It was observed that during the increase in temperature to 200 °C, there was no evidence of crystalline calcium silicate hydrate formation in the products (Figure 8). Consequently, intense diffraction peaks of unreacted quartz {SiO_2_ (PDF 00-046-1405; *d*-spacing—0.461; 0.335; 0.246 nm)} and portlandite {Ca­(OH)_2_ (PDF 00-044-1481; *d*-spacing—0.492; 0.263; 0.193 nm)} were identified. Prolonging the synthesis duration up to 4 h positively affected the interaction between raw materials because portlandite fully reacted, while the area of the main diffraction peak (*d-*spacing—0.335 nm) characteristic of quartz decreased from 11.5 (0 h) to 1.5 (4 h) (Figure 8). It should be mentioned that traces of the latter compound were still present in the sample even after 72 h of isothermal treatment. However, the area of the quartz main peak was only 0.31 cps. This can be explained by the low solubility of quartz compared to other silica compound varieties, such as amorphous silica or colloidal silica gel.^48^

**Figure 8:**
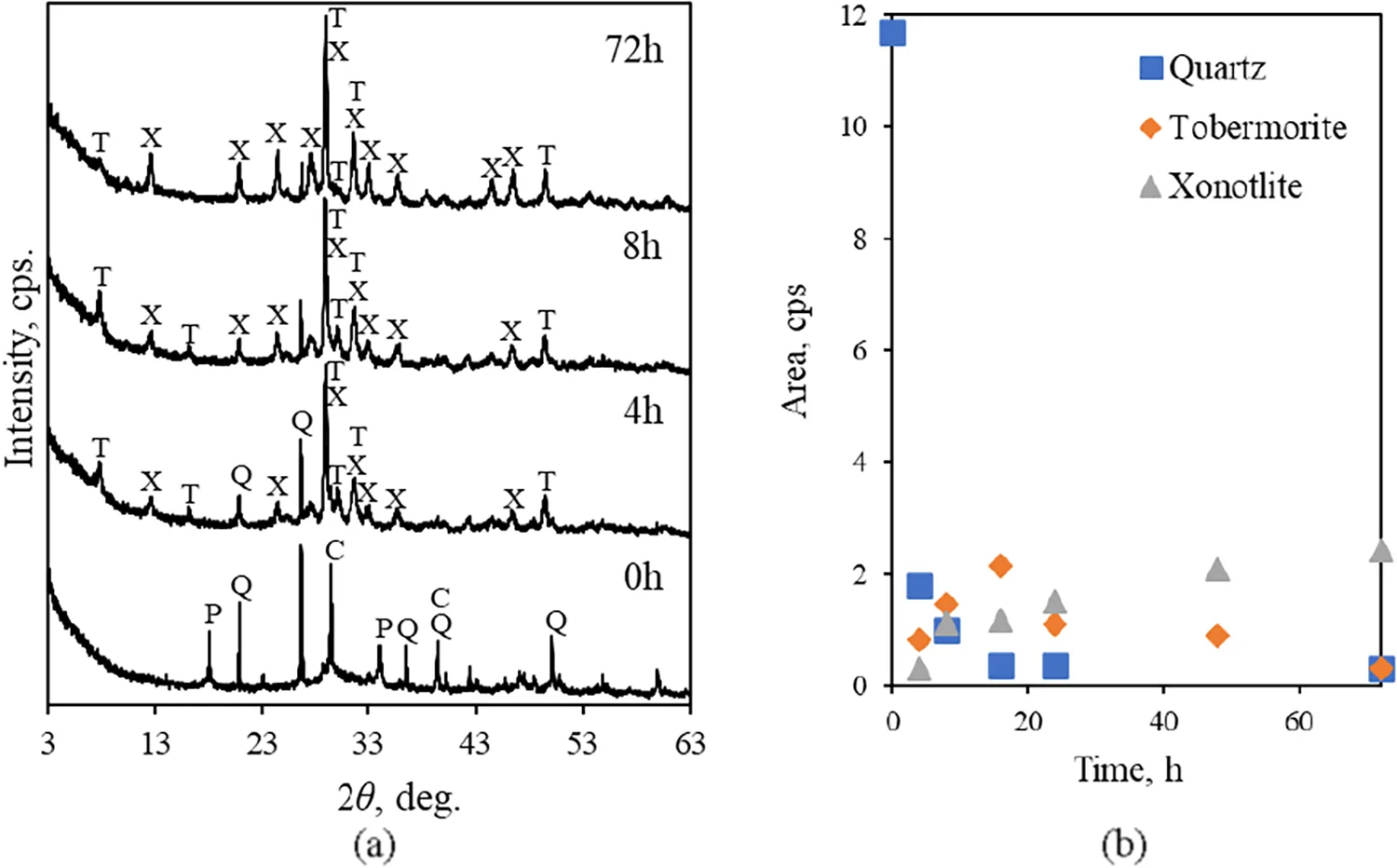
(a) XRD curves of synthesis products. Indices: Q—quartz, P—portlandite, C—calcite, T—tobermorite, X—Xonotlite. (b) Quartz, tobermorite, and Xonotlite diffraction peak area dependency on synthesis time.

In addition, calcium carbonate {CaCO_3_ (PDF 04-012-8072; *d*-spacing—0.304; 0.210; 0.191 nm)} formed during the increase in temperature to 200 °C (Figure 8). Calcium carbonate appeared in the sample due to the carbonation of calcium-containing compounds during synthesis or drying in the air-conditioned chamber (60 °C, 24 h).^49^

Low-basicity crystalline calcium silicate hydrates, Xonotlite {Ca_6_Si_6_O_17_(OH)_2_ (PDF 00-023-0125; *d*-spacing—0.705; 0.324; 0.195 nm)} and tobermorite {Ca_5_(OH)_2_Si_6_O16·4H_2_O (PDF 00-019-1364; *d*-spacing—1.134; 0.549; 0.298 nm)}, dominated in the products after 4–72 h of hydrothermal treatment. It should be mentioned that the molar ratio of CaO/SiO_2_ in tobermorite is 0.83, which is lower than that of the raw mixture (CaO/SiO_2_ – 1). According to the scientific literature, tobermorite is a flexible crystalline calcium silicate hydrate because it can form in mixtures with various molar ratios of CaO/SiO_2_, ranging from 0.66 to 1.5.^50–52^

It was calculated that the tobermorite diffraction peak (*d*-spacing—1.134 nm) area increased from 0.83 to 2.15 by extending the duration of synthesis from 4 to 16 h. Further extension of the duration led to a decrease in the tobermorite diffraction peak area, and after 72 h, the diffraction peak area was equal to 0.3.

Meanwhile, Xonotlite showed different behavior, because the area of its main diffraction peak (*d*-spacing—0.324 nm) increased only from 0.25 to 1.18 when the duration was increased from 4 to 16 h. The area increased twice (to 2.42) by extending the synthesis duration to 72 h. These results suggest that tobermorite became metastable and recrystallized into Xonotlite upon prolonging the synthesis duration.^53^

In summary of the XRD results, the formation of calcium silicate hydrates during hydrothermal synthesis at 200 °C can be described by the following sequence

$$CaO+{SiO}_{2}+H_{2}O\overset{0h}{\to }\begin{array}{l} Portlandite↑\\ Calcite↓\\ Quartz↓ \end{array}\overset{4-16h}{\to }\begin{array}{l} Tobemorite↑\\ Xonotlite↓\\ Quartz↓ \end{array}\overset{24-72h}{\to }\begin{array}{l} Tobemorite↓\\ Xonotlite↑\\ Quartz↓ \end{array}$$

The products produced after 16 and 72 h of hydrothermal synthesis were characterized using FT-IR analysis (Figure 9). It was observed that in both FT-IR curves, similar absorption bands were identified, whose intensities varied insignificantly with the duration of the synthesis. The characteristic bands of tobermorite and Xonotlite were identified in the higher and middle frequency regions (600–1200 cm^–1^): asymmetric stretching vibrations of Si–O–Si in Q^3^ silicon sites at 1202 cm^–1^, asymmetric stretching vibrations of Si–O–Si in Q^2^ silicon sites at ∼1060 cm^–1^, stretching vibrations of O–SiO– at ∼961 cm^–1^, symmetric stretching vibrations of Si–O–Si at 670–739 cm^–1^, and deformation vibrations of Si–O–Si at 607–627 cm^–1^. These vibrations confirm the formation of tobermorite and Xonotlite in the product (Table 3).^54,55^

**Figure 9:**
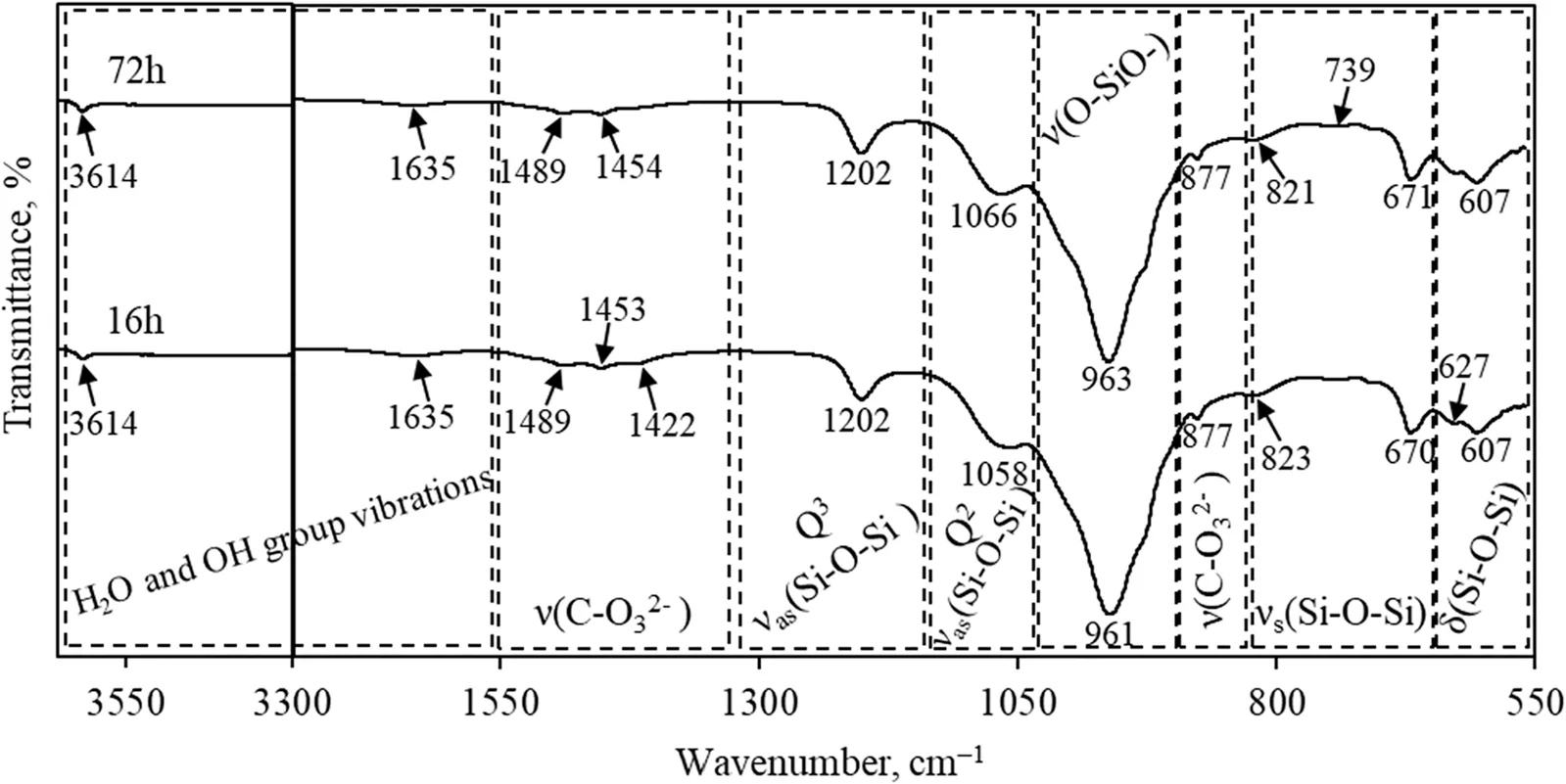
FT-IR curves of synthesis products.

**Table 3:** Absorption Bands Characteristic of Xonotlite, Tobermorite, and Calcite

vibration	wavenumber, cm^–1^	Xonotlite	tobermorite	CaCO_3_
ν(OH) ν(H_2_O)	3614	+		
	3395	+		
	1635	+	+	
ν(C=O)	1489			+
	1453			+
	1442			+
ν_as_(Si–O–Si)	1202	+	+	
	1058	+	+	
	1066		+	
ν(O-SiO−)	961	+	+	
	963	+	+	
ν(C–O_3_^2–^)	877			+
ν_s_(Si–O–Si)	823	+	+	
	670	+	+	
	671	+	+	
δ(Si–O–Si)	627	+		
	607	+		

Absorption bands characteristic of stretching vibrations of the CO_3_^2–^ group were observed in the 1400–1500 cm^–1^ region and at 877 cm^–1^. This indicates that the synthesis products may contain a small amount of calcium carbonate, which was not identified in the XRD curves.^55^ Also, these bands may be caused by CO_2_ in the environment.

Additionally, in both FT-IR curves, a small absorption band at 1635 cm^–1^ was observed, which can be attributed to the bending vibration of coordinated water (H–O–H), indicating that the products contain interlayer water.^56^

STA analysis confirmed the previously discussed results (Figure 10a). The first endothermic effect was observed at 135 °C, indicating the removal of moisture and crystalline water from the product, with a corresponding mass loss of 6.63%. The second endothermic effect was observed at 687 °C, which correlated with the presence of calcium carbonate in the product. According to TGA data, the mass loss was 1.48%, which indicated that the synthesis product contained 3.36 wt % of calcium carbonate. This is in good agreement with the FT-IR data, where absorption bands characteristic of CO_3_^2–^ group vibrations were identified (Table 3). The exothermic effect at 830 °C can be attributed to the recrystallization of calcium silicate hydrates into wollastonite (CaSiO_3_).^57^ It was calculated that during this exothermic effect, the heat flow was equal to 35.00 J/g.

**Figure 10:**
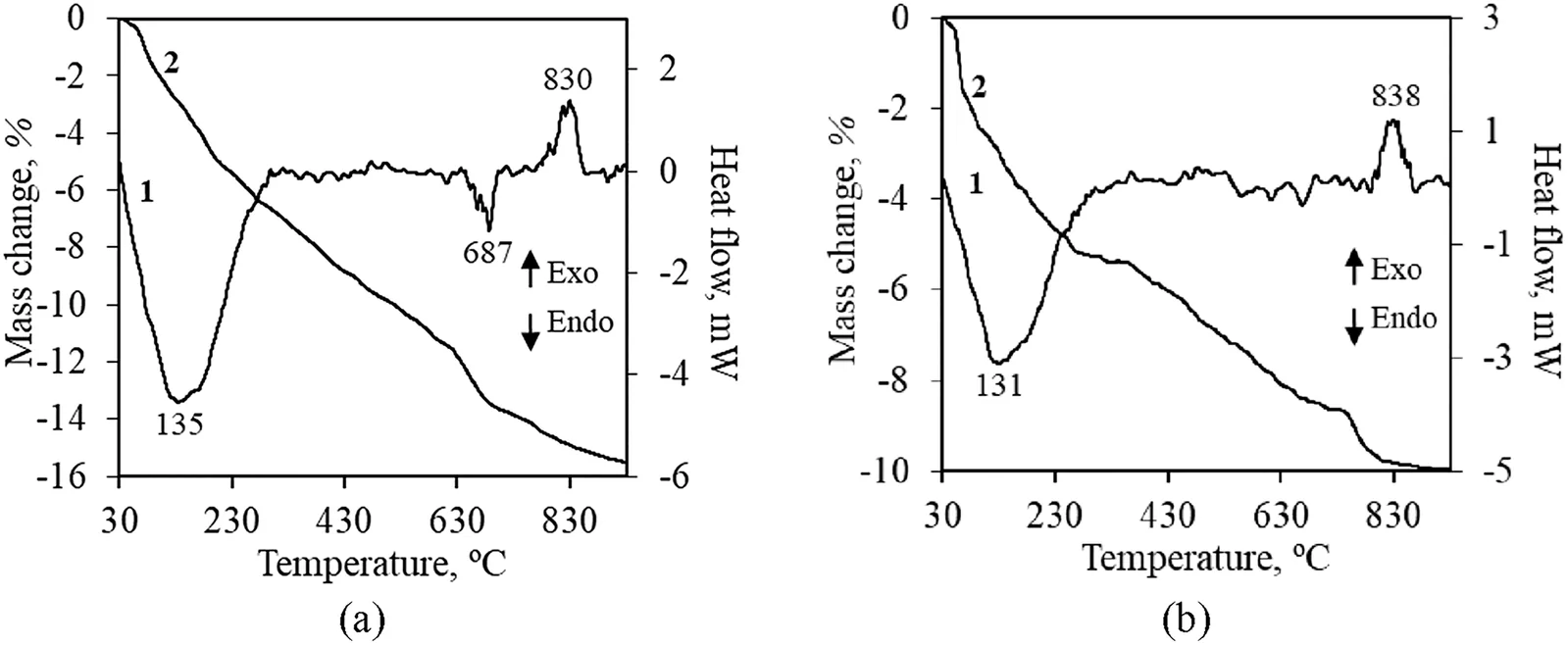
STA curves of synthesis products. Indices: 1—DSC, 2—TGA: (a) 16 h; (b) 72 h.

It was determined that the synthesis duration influenced the profile of the STA curves (Figure 10). Only one endothermic effect was observed in the product of the 72 h hydrothermal synthesis, indicating the removal of adsorbed and crystalline water from the product, with a corresponding mass loss of 5.29% (Figure 10b). During this process, the mass loss was lower due to a higher amount of Xonotlite and a smaller amount of tobermorite, which also meant a smaller amount of water in the product. An exothermic effect was observed at 828 °C, indicating that calcium silicate hydrates recrystallized into wollastonite, as mentioned before. The heat flow for the aforementioned process was calculated to be 33.29 J/g, which was slightly lower compared to the sample obtained after 16 h.

It should be mentioned that neither Xonotlite nor tobermorite exhibited a distinct thermal effect in the temperature range of 250–850 °C characteristic of these phases, because their dehydration proceeds gradually, as seen in the TGA data (Figure 9). It was calculated that the total mass loss for the product of a 16 h long synthesis was 16.01%, while the total mass loss for the product of a 72 h long synthesis was 10.03%. The mass loss correlates with the amount of water in tobermorite (Ca_5_(OH)_2_Si_6_O16·4H_2_O) and Xonotlite (Ca_6_Si_6_O_17_(OH)_2_), which confirms that by prolonging synthesis duration, tobermorite recrystallized into Xonotlite.

#### 3.2.2 Wollastonite Formation

The XRD results showed that tobermorite and Xonotlite were the main crystalline calcium silicate hydrates formed during hydrothermal synthesis (Figure 8a). The most intense diffraction peaks characteristic of tobermorite and Xonotlite were obtained after 16 and 72 h, respectively. Thus, these samples were used as wollastonite precursors. Based on the dominant CSH phase in the products and synthesis duration, the sample obtained after 16 h of synthesis was labeled as T–16, while the sample obtained after 72 h was labeled as X–72.

It was determined that tobermorite dehydrated, forming semicrystalline calcium silicates during the calcination of the T–16 sample at 400 °C (Figures 11a and 12a).^58^ This is in good agreement with the results presented in the scientific literature.^57^ Xonotlite remained stable up to 700 °C, as no significant change in its diffraction peak area was observed (Figure 11a). At a higher temperature (800 °C), Xonotlite and semicrystalline calcium silicates recrystallized into β-wollastonite (Figures 11a and 12a). This confirmed the nature of the exothermic effect observed in the STA curves (Figure 10). It was calculated that the area of the main diffraction peak characteristic of β-wollastonite increased from 11.45 to 15.66 with increasing calcination temperature from 800 to 1100 °C. The higher calcination temperature (1200 °C) led to the formation of pseudowollastonite, and as a result, the area of the main diffraction peak characteristic of β-wollastonite decreased to 4.1. At 1300 °C, pseudowollastonite was the only product identified in the XRD patterns of the T–16 sample.

**Figure 11:**
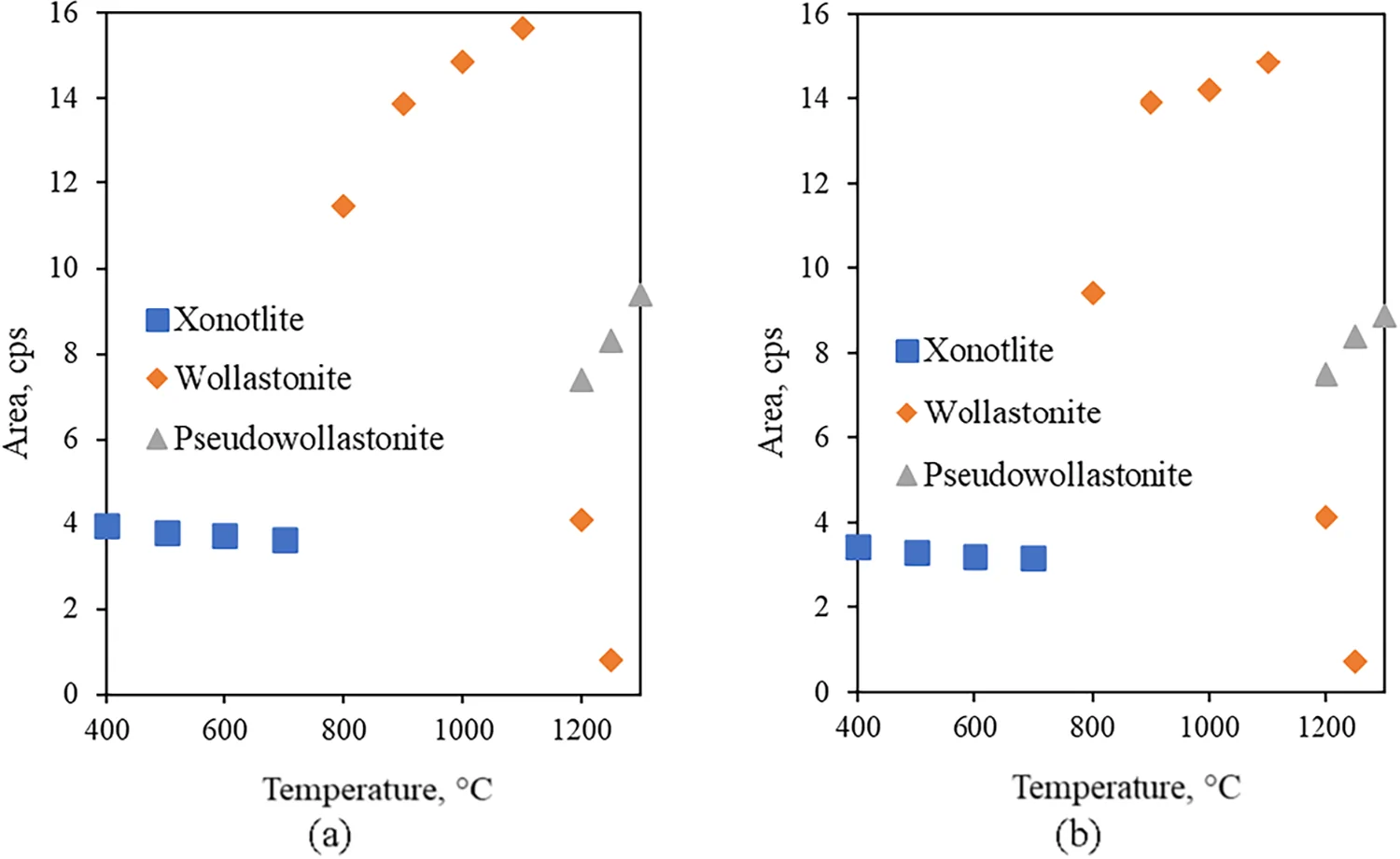
Diffraction peak area dependency on calcination products: (a) T–16; (b) T–72.

**Figure 12:**
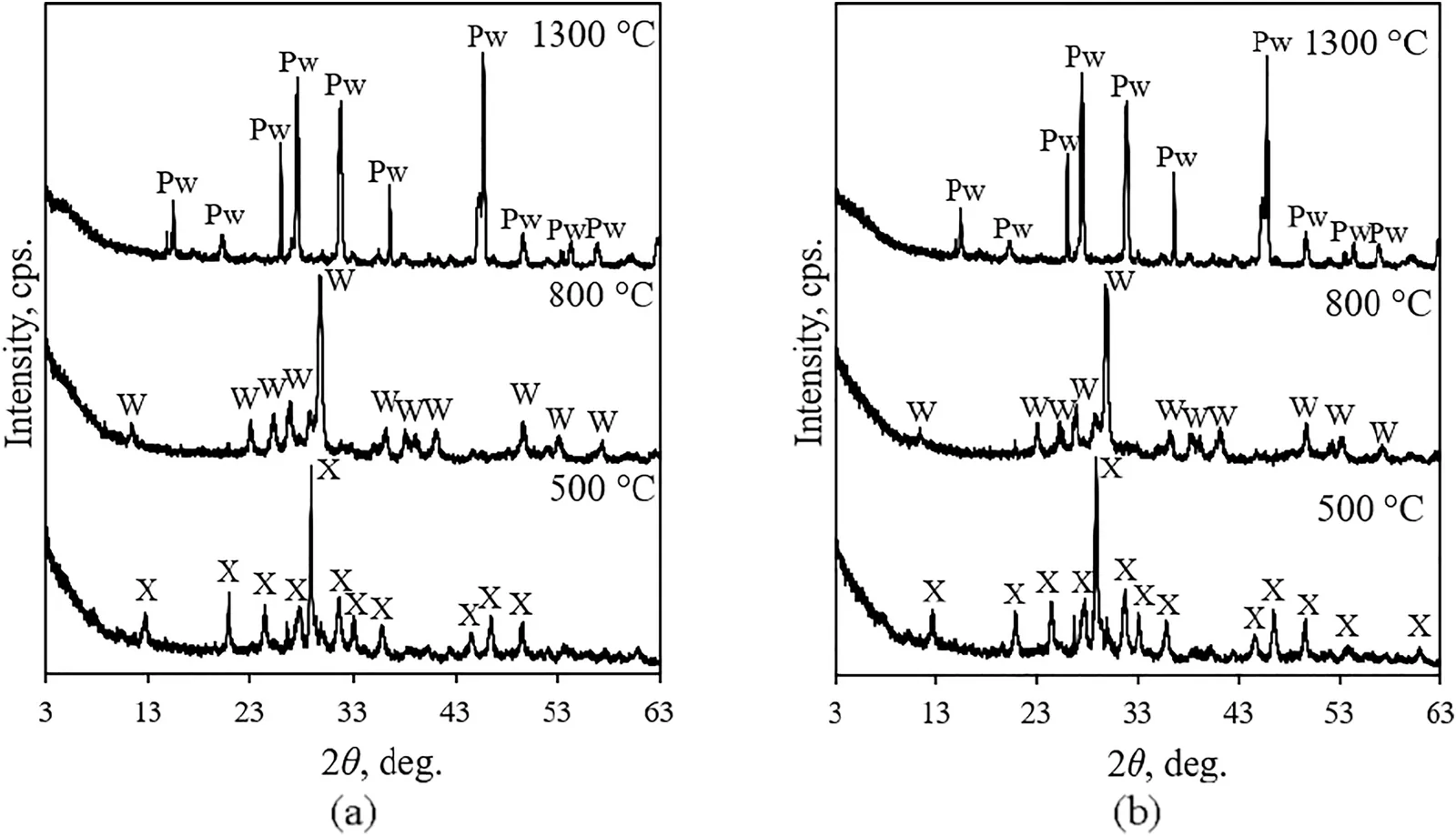
XRD curves of calcinated T–16 (a) and X–72 (b) samples. Indices: X—Xonotlite; W—wollastonite; Pw—pseudowollastonite.

The formation of wollastonite proceeded similarly during the calcination of the X–72 sample (Figures 11 and 12). Xonotlite remained stable up to 700 °C and at a higher temperature (800 °C) recrystallized into β-wollastonite. As in the previous case (sample T–16), the area of the main diffraction peak characteristic of wollastonite increased with an increase in temperature. However, wollastonite formed during the calcination of the X–72 sample was characterized by a lower area of the main diffraction peak (*I* – 14.85) compared to the T–16 sample (*I* – 15.66). Further calcination (1200 °C) led to the formation of pseudowollastonite, which became the only product at 1300 °C.

Thus, the two-step synthesis allowed the obtaining of pure β-wollastonite (800–1000 °C) and α-wollastonite (1300 °C), while during one-step synthesis, wollastonite was always obtained in a mixture with other calcium silicates.

#### 3.2.3 Morphology of Synthetic Samples

For practical applications, the X–72 sample calcined at 500 and 1000 °C was used. The X–72 sample was chosen because, after 72 h of hydrothermal synthesis, Xonotlite, with only impurities of tobermorite, was formed. Meanwhile, the calcination temperature of 500 °C was chosen due to the dehydration of Xonotlite without losing its crystallinity, while 1000 °C was chosen because β-wollastonite was formed. In the next part of the research, the samples were labeled based on the dominant phase after hydrothermal synthesis (Xonotlite) and calcination temperature (500 and 1000 °C).

The density of the samples was determined by a pycnometer using He gas. It was found that the density of the X–72–500 sample was equal to 2.166 g/cm^3^, while after calcination at 1000 °C, it increased to 2.302 g/cm^3^ for the X–72–1000 sample. The increase in density can be related to particle agglomeration and decreased porosity due to the beginning of sintering.

Nitrogen adsorption and desorption experiments at 77 K were performed to evaluate the porosity and specific surface area of the samples (Figure 13). The adsorption isotherms of both samples can be classified as type II, which, according to the IUPAC classification, is characteristic of nonporous or macroporous materials. Such isotherms cannot be used for reliable pore volume determination; however, they can be used to determine the specific surface area by applying the BET method.^59^

**Figure 13:**
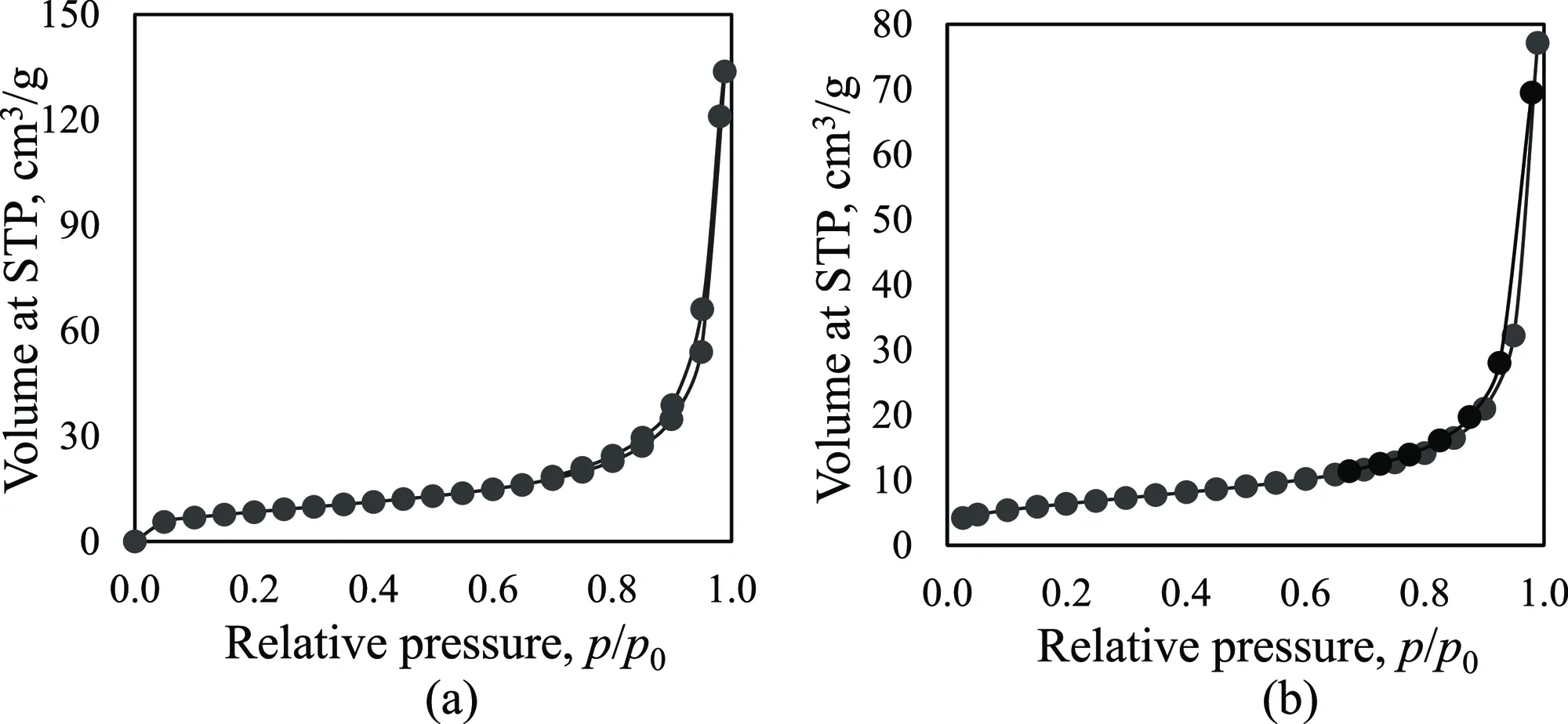
Adsorption and desorption isotherms of X–72–500 (a) and X–72–1000 (b) samples.

The BET constant, *C*_BET_, which is related to the formation of a monolayer, was calculated to be 50 and 113 for X–72–500 and X–72–1000, respectively. These values fall within the range of 50–150, confirming that the BET equation can be applied. The specific surface area of X–72–500 and X–72–1000 was calculated to be 31.585 and 22.979 m^2^/g, respectively. These results are in good agreement with the density measurements, since the X–72–1000 sample showed a higher density and lower specific surface area, which can be associated with particle agglomeration and partial sintering at higher temperature.

Particle size distribution analysis using a CILAS LD 1090 analyzer was additionally performed to determine the particle size distribution of the synthesis products (Figure 14a,b). It was determined that the particles of both samples were distributed over a wide range, from approximately 1 to 50 μm. The X–72–500 sample was characterized by smaller particles: diameter at 10%—0.58 μm, at 50%—11.86 μm, at 90%—36.68 μm, and mean diameter—15.96 μm. In comparison, the X–72–1000 sample showed larger particles: diameter at 10%—0.95 μm, at 50%—13.64 μm, at 90%—42.88 μm, and mean diameter—18.74 μm. This increase in particle size can be explained by the beginning of particle sintering at higher calcination temperature.

**Figure 14:**
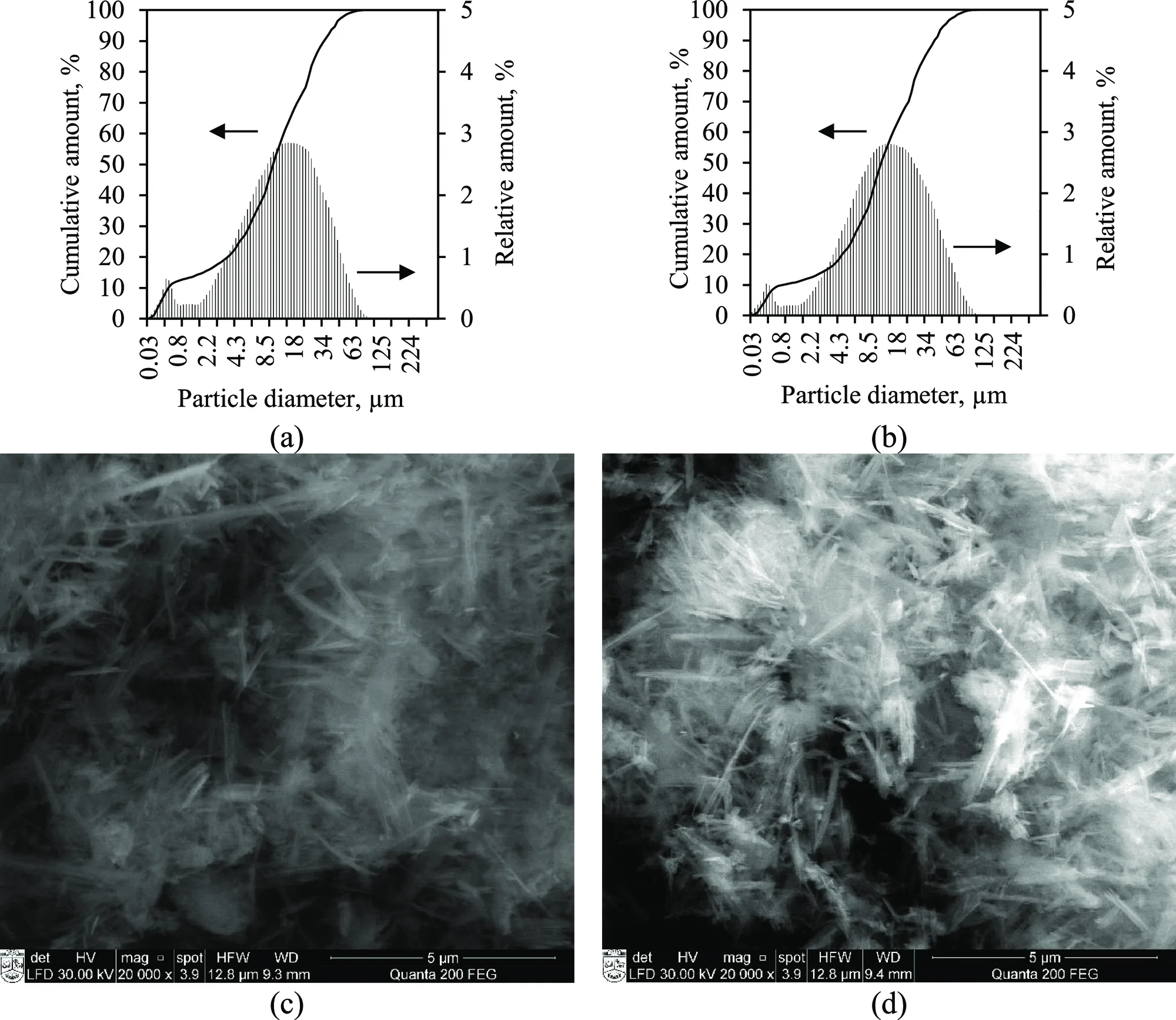
Particle size distribution (a, b) and SEM micrographs (c, d) of X–72–500 (a, c) and X–72–1000 (c, d).

Finally, SEM analysis was performed to visually evaluate the morphology of the particles (Figure 14 c,d). SEM micrographs showed that both samples consist of fiber- and needle-like structures. However, after calcination at 1000 °C, the morphology became more pronounced, and the particles appeared coarser and more agglomerated. This observation is consistent with the particle size distribution, density, and BET results, which indicate increased particle agglomeration, reduced specific surface area, and partial sintering in the X–72–1000 sample. Besides, the data presented in Table 4 show that the properties of wollastonite obtained from secondary raw materials vary depending on the raw materials and synthesis conditions. The X–72–1000 sample obtained from SWC and quartz sand had a density of 2.302 g/cm^3^ and low porosity. A similar density of 2.2 g/cm^3^ was reported for wollastonite produced from cement bypass dust and silica fume,^62^ while the material obtained from cement bypass dust and ceramic sludge had a lower density and considerably higher porosity.^63^

**Table 4:** Comparison of Wollastonite Produced from Secondary Raw Materials

calcium source	silica source	density (g/cm^3^)	porosity	BET surface area (m^2^/g)	morphology	ref.
SWC	quartz sand	2.302	low	31.585	needle-fiber-like	this work
eggshell	fumed silica				β-wollastonite (1000 °C) → pseudowollastonite (1250 °C); nanoscale 25–46 nm	^60^
eggshell	rice husk ash				single-phase β-wollastonite	^61^
cement bypass dust	silica fume	2.2 (at 1170 °C)	8% apparent (at 1170 °C)		needle/acicular (900–1050 °C) → anhedral (1100–1170 °C)	^62^
cement bypass dust	ceramic sludge	1.28–1.57	43–57% open		porous, wollastonite + gehlenite + cristobalite	^63^
granite waste	marble waste			6	nanoscale (<100 nm)	^64^
limestone	silica sand	2.93	low	3.23	dense α-wollastonite	^65^

The specific surface area of the X–72–1000 sample was 22.979 m^2^/g, which was higher than the values reported for products obtained from granite and marble waste^64^ and from limestone and silica sand.^65^ Differences were also observed in the morphology of the synthesis products. The X–72–1000 sample consisted mainly of needle- and fiber-like particles, while a similar acicular morphology was reported for wollastonite obtained from cement bypass dust and silica fume at 900–1050 °C.^62^ In other studies, nanoscale particles^60,64^ or porous structures containing several crystalline phases^63^ were obtained. These results indicate that SWC can be used as a calcium-containing raw material for the synthesis of wollastonite with a relatively high specific surface area and characteristic needle- and fiber-like morphology.

### 3.3 Photocuring Kinetics

The resin **50B/50H** containing 50 wt % of BisGMA and 50 wt % of HEMA was selected as a comparative resin, which is frequently used in copolymerization studies.^66^ BisGMA provides high cross-link density, whereas HEMA acts as a low-viscosity reactive diluent, reducing resin viscosity and improving monomer mobility during photopolymerization. 25 wt % of CMA was incorporated into the resin **50B/25H/25C** to partially replace HEMA in order to introduce an aromatic moiety and additional ester groups into the cross-linked network, thereby influencing network rigidity and enabling transesterification-based dynamic bond exchange. Wollastonite (X–72–1000) and Xonotlite (X–72–500) were incorporated at 10 and 20 wt %, representing typical filler amounts used in polymer composites,^67^ to evaluate their influence on viscosity and curing kinetics. Higher filler contents were not used to avoid viscosity increase and possible reduction of self-welding properties caused by restricted network mobility. Rheological characterization was performed to evaluate the effect of monomer and filler amount on curing behavior. Rheological analysis is an essential tool for evaluating the processability and curing behavior of photocurable resin systems, particularly for coating applications. Resin viscosity shows flow behavior and filler dispersion prior to curing, while rheological measurements performed during irradiation provide valuable insight into the formation and evolution of the cross-linked polymer structure.^68^ The viscosity values of the prepared resins are presented in Table 5. Among the formulations without filler, the resin **50B/25H/25C** containing CMA exhibited higher viscosity than the resin **50B/50H** without CMA, which can be attributed to the higher viscosity of the CMA monomer compared to HEMA. The addition of inorganic fillers resulted in an increase in viscosity, which depended on filler content. Resins **10W** and **10X** containing 10 wt % filler showed an increase in viscosity, whereas a higher viscosity was observed with 20 wt % of filler, indicating increased resistance to flow due to particle–particle interaction.^69^ At the same filler amount, wollastonite-filled resins exhibited higher viscosity than Xonotlite-filled resins due to differences in particle morphology and particle-resin interactions.^70^ The evolution of *G*′ modulus during UV/vis irradiation is shown in Figure 15, and the corresponding characteristic values are summarized in Table 5. Upon UV/vis exposure, all resins exhibited a rapid increase in *G*′, which is characteristic of methacrylate-based photocurable systems and indicates efficient photopolymerization under the applied irradiation conditions.^71^ All resins exhibited the same gel point (*t*_gel_ = 1 s), indicating that neither CMA incorporation nor filler addition significantly affected the onset of cross-linking under the applied irradiation conditions. Among the resins without filler, the resin **50B/25H/25C** containing CMA exhibited a higher *G*′ modulus compared to the resin **50B/50H**. This behavior can be attributed to the presence of the aromatic moiety in CMA, which contributes to increased network rigidity and may also be influenced by the additional methacrylate group contributing to network formation. The incorporation of inorganic fillers into the CMA-containing resin **50B/25H/25C** resulted in a decrease in the *G*′ modulus compared to the corresponding resin without filler. Moreover, the *G*′ modulus decreased with increasing filler amount, indicating that the presence of solid particles affects network development during photopolymerization and the resulting modulus of the cured system. Although filler addition increased the viscosity of the uncured resins due to particle-induced flow restriction, the final rigidity depended mainly on polymer network formation, filler dispersion, and filler-resin interactions rather than the initial viscosity of the resins. At the same filler amount, wollastonite-filled resins exhibited higher *G*′ modulus values than Xonotlite-filled resins. This behavior can be related to differences in particle morphology and particle-resin interactions. The complex viscosity values also decreased upon filler incorporation, consistent with the observed reduction in *G*′ modulus. The *G*″ modulus increased in Xonotlite-filled resins compared to the formulation without filler, indicating enhanced energy dissipation in the presence of inorganic particles during photopolymerization. Among the resins without filler, the incorporation of CMA resulted in a reduction of 7.5% in shrinkage compared to the resin **50B/50H**. This reduction can be attributed to the presence of the aromatic moiety in CMA and its influence on network structure, which limits volume shrinkage during curing. The addition of inorganic fillers reduced shrinkage compared to the resin **50B/50H**, while shrinkage values remained comparable to those of the CMA-containing resin **50B/25H/25C** without filler. This indicates that the presence of inorganic filler influences the curing behavior of the resin, leading to reduced volume change during polymerization. Increasing filler amount did not lead to further shrinkage reduction, and no significant differences were observed between wollastonite- and Xonotlite-filled resins, suggesting that shrinkage is influenced by resin composition rather than filler type. The rheological measurement results demonstrate that resin composition determines storage modulus development during curing, as the incorporation of CMA results in a higher *G*′ modulus compared to the *G*′ modulus of the resin **50B/50H**. In contrast, the incorporation of inorganic fillers increases viscosity and decreases *G*′ modulus, while Xonotlite-filled resins exhibit a higher *G*″ modulus than wollastonite-filled resins. Overall, both monomer structure and filler type influence the rheological properties during photopolymerization.

**Table 5:** Rheological Parameters of the Prepared Resins

sample	*t*_gel,_s	storage modulus *G*′, MPa	loss modulus *G*″, MPa	complex viscosity, MPa·s	shrinkage, %
**50B/50H**	1 ± 0	17.74 ± 1.74	1.81 ± 0.71	568.10 ± 7.24	22.5 ± 3.5
**50B/25H/25C**	1 ± 0	19.25 ± 0.05	2.74 ± 0.22	617.69 ± 2.06	15.0 ± 0.0
**10W**	1 ± 0	18.37 ± 1.10	2.30 ± 0.28	595.12 ± 9.63	16.5 ± 0.5
**20W**	1 ± 0	18.07 ± 1.07	1.99 ± 0.31	578.45 ± 4.90	17.0 ± 1.0
**10X**	1 ± 0	17.34 ± 0.16	4.05 ± 0.37	568.74 ± 0.64	16.0 ± 1.0
**20X**	1 ± 0	16.49 ± 0.29	3.31 ± 1.02	535.98 ± 5.25	16.5 ± 0.5

**Figure 15:**
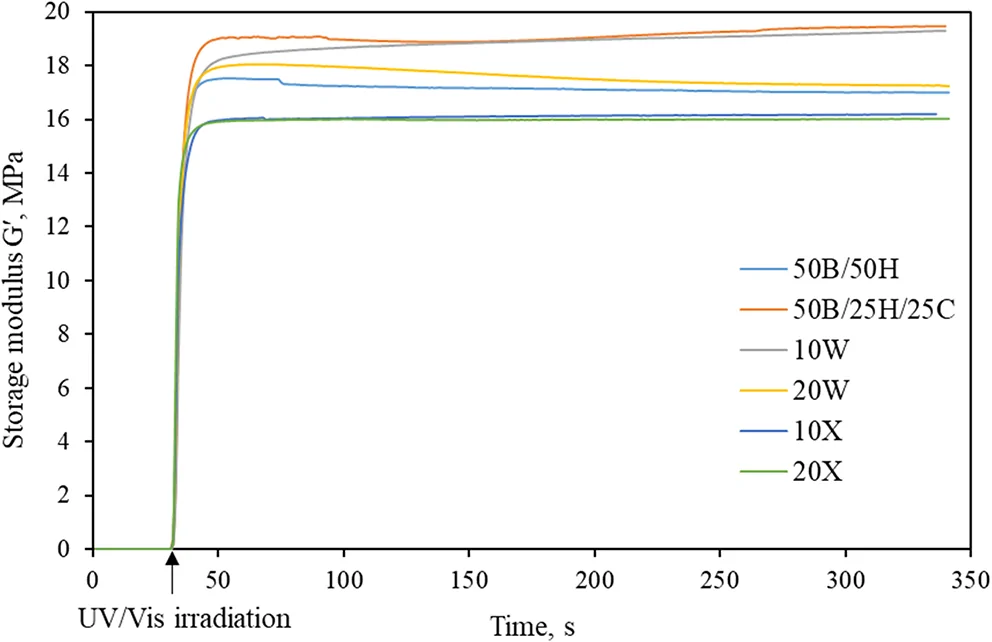
Storage modulus *G*′ curves versus irradiation time of resins.

### 3.4 Self-Welding

In vitrimer networks, dynamic covalent bond exchange activated at elevated temperature enables network rearrangement and interfacial reconnection across damaged regions without the use of external adhesives, allowing partial recovery of mechanical characteristics after fracture.^32^ The efficiency of this process depends on the mobility of network segments and the presence of inorganic fillers, which may influence chain mobility and interfacial contact during the self-welding process.^38^ The capability of a coating to restore properties after damage can prolong service life and maintain its protective function. The self-welding ability of the cured resins was evaluated by joining two previously photopolymerized and cut specimens into a single sample at 180 °C, as shown in Figure 16.

**Figure 16:**
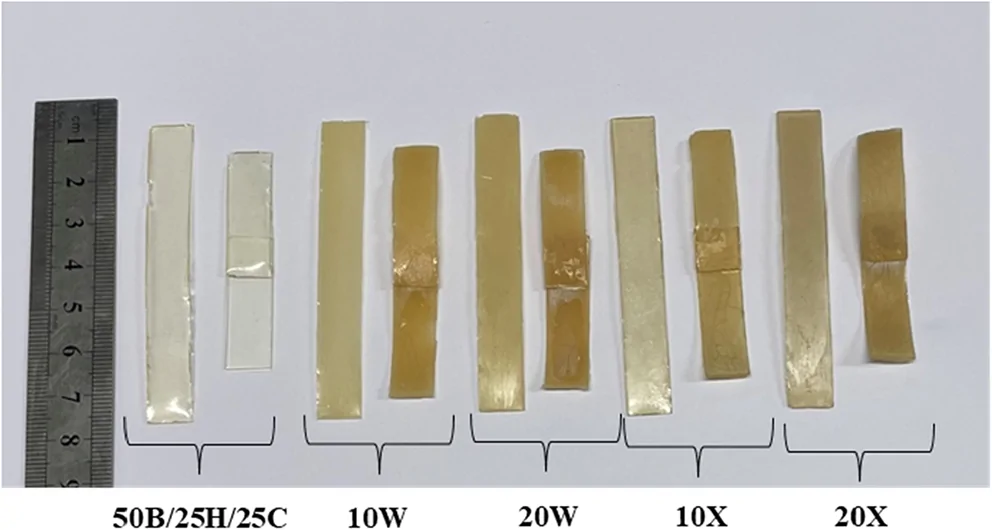
Photograph of the samples obtained after UV/vis curing and after self-welding at 180 °C.

To evaluate the efficiency of the self-welding process, mechanical properties were measured before and after self-welding. The mechanical characteristics of the prepared resins before and after the self-welding process are presented in Figure 17. For the sample **50B/25H/25C** without filler, the tensile strength decreased from 14.17 to 10.00 MPa after self-welding, corresponding to a reduction of 30%, which indicates a self-welding efficiency of 70% based on tensile strength recovery. The elongation at break decreased from 4.39 to 2.50%, while Young’s modulus decreased from 242.25 to 120.00 MPa (50% reduction). These results indicate partial recovery of strength, although stiffness was significantly reduced after welding. The incorporation of inorganic fillers resulted in higher Young’s modulus values before self-welding. For example, the Young’s modulus increased from 242.25 MPa for the sample **50B/25H/25C** without filler to 352.81 and 954.45 MPa for samples **10W** and **20W**, respectively, and to 880.48 and 968.93 MPa for samples **10X** and **20X**, confirming the stiffening effect of both fillers. However, after self-welding, a decrease in tensile strength was observed for all samples with fillers. The tensile strength of the sample **10W** decreased from 5.26 to 0.93 MPa (self-welding efficiency of 18%), while that of the sample **10X** decreased from 12.00 to 0.49 MPa (self-welding efficiency of 4%). Similarly, Young’s modulus decreased from 352.81 to 109.65 MPa for the sample **10W** and from 880.48 to 346.86 MPa for the sample **10X**. Increasing filler amount up to 20 wt % maintained high stiffness before welding but did not significantly improve strength recovery after welding, as tensile strength decreased from 5.72 to 1.99 MPa for the sample **20W** (a self-welding efficiency of 35%) and from 2.40 to 0.90 MPa for the sample **20X** (a self-welding efficiency of 3%). In addition, Young’s modulus decreased from 954.45 to 489.68 MPa for the sample **20W** and from 968.93 to 558.35 MPa for the sample **20X**, indicating that stiffness was partially retained. For all samples, elongation at break decreased after welding, indicating reduced elasticity at the self-welded interface. For the sample **50B/25H/25C**, elongation decreased by 43%. A higher reduction was observed for the samples **10W** (68%) and **10X** (32%), while the sample **20X** showed a decrease of about 34%. In contrast, the sample **20W** exhibited only a minor reduction in elongation (2%), indicating a limited change in elasticity at a higher wollastonite amount.

**Figure 17:**
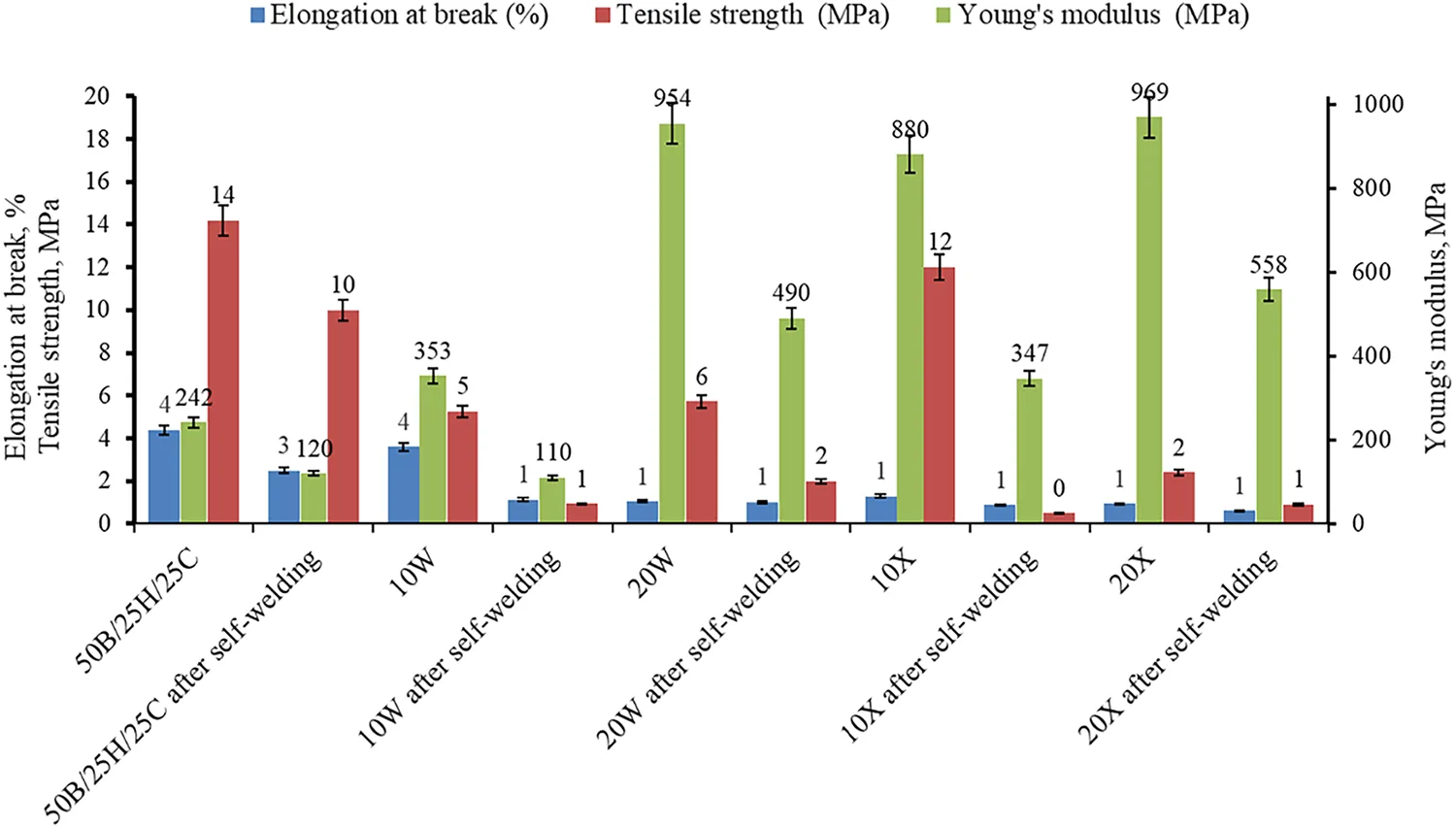
Mechanical characteristics of the samples obtained after UV/vis curing and after self-welding at 180 °C.

The FT-IR spectra recorded before and after self-welding (Figure 18) showed a reduced intensity of the O–H group (3450 cm^–1^) and the C=O group signals (1715 cm^–1^). These changes suggest modifications of hydroxyl and ester groups during thermal treatment, consistent with dynamic bond exchange. However, differences in hydrogen bonding can also contribute to intensity change.^65^ Importantly, no new absorption bands were observed, indicating that the chemical structure of the network remained unchanged. This confirms that the recovery of mechanical properties is associated with dynamic bond exchange and network rearrangement rather than additional reactions of residual functional groups.

**Figure 18:**
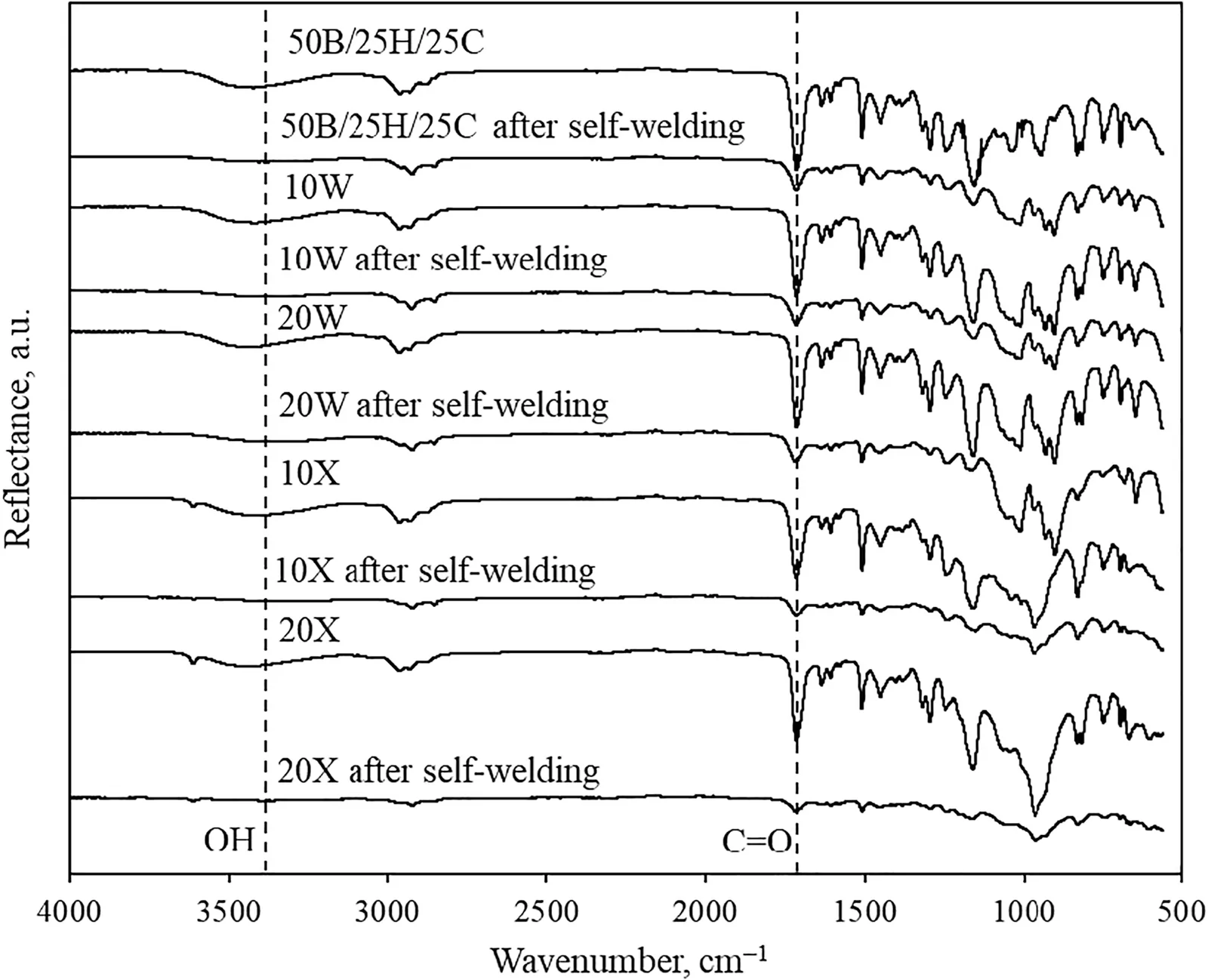
FT-IR spectra before and after self-welding.

Although self-welding efficiency was lower in the samples with fillers compared to the sample without filler, partial recovery of mechanical properties was still observed, confirming that self-welding occurred. The reduced recovery suggests that the presence of particles restricts segmental mobility and limits the extent of dynamic bond exchange across the interface. In coatings, the incorporation of inorganic fillers results in an increase in Young’s modulus of the cured resin, which may enhance resistance to mechanical deformation. At the same time, the observed partial recovery of mechanical characteristics demonstrates that dynamic bond exchange remains in the presence of fillers. Thus, appropriate control of filler amount allows the combination of enhanced stiffness with retained self-welding capability, which is advantageous for coatings exposed to repeated mechanical damage.

## 4 Conclusions

This study demonstrates that calcium-rich waste from the sugar industry can be effectively used as a precursor for the synthesis of calcium silicates, although the synthesis method determines the phase composition and product quality. Direct one-step calcination of calcium-rich waste and quartz mixture (CaO/SiO_2_ = 1.00) at 400–1300 °C resulted in the formation of complex phase compositions dominated by larnite and rankinite at lower temperatures (900–1100 °C), wollastonite at temperatures ≥1100 °C. This indicates the limited suitability of this method for the production of high-purity wollastonite.

A two-step method combining hydrothermal treatment at 200 °C with subsequent calcination ensured a controlled phase formation. A 16 h hydrothermal synthesis resulted mainly in the formation of tobermorite, while an extension of the duration to 72 h promoted the formation of Xonotlite as the dominant phase. Calcination of tobermorite and Xonotlite resulted in the formation of phase-pure β-wollastonite at 800–1000 °C and α-wollastonite at 1300 °C, indicating a significantly lower effective wollastonite formation temperature compared to the one-step process.

Waste-derived calcium silicates can be effectively integrated into photocurable vitrimer systems as functional fillers, enabling simultaneous improvement of mechanical rigidity and the retention of self-welding capability. This integrated material design strategy supports the development of sustainable, repairable coating systems and highlights the potential of combining waste valorization with advanced polymer network concepts. Photocurable, catalyst-free vitrimer composites based on bisphenol A glycerolate dimethacrylate, 2-hydroxyethyl methacrylate, and cinnamyl methacrylate reinforced with calcium silicate fillers (wollastonite and Xonotlite) were successfully synthesized, and their rheological and self-healing properties were investigated. Partial substitution of 2-hydroxyethyl methacrylate with cinnamyl methacrylate introduced aromatic moieties and additional ester groups into the cross-linked network, leading to increased rigidity of the cured material and reduced polymerization shrinkage compared to those of the bisphenol A glycerolate dimethacrylate and 2-hydroxyethyl methacrylate resin. An increasing filler amount resulted in higher viscosity prior to curing and a decrease in the storage modulus of the cured cinnamyl methacrylate-containing network. Differences between wollastonite- and Xonotlite-filled resins were attributed to variations in particle morphology and particle-resin interactions. All investigated resins demonstrated self-welding capability after thermal treatment, confirming the presence of a dynamic covalent bond exchange within the network. At higher filler amounts, restricted segmental mobility and limited interfacial rearrangement further decreased the restoration of mechanical properties, although the self-welding ability was still retained. The combination of rapid photopolymerization, enhanced rigidity after curing, and thermally activated self-welding indicates that these vitrimer composites are suitable candidates for coating systems, where mechanical durability and repairability are required.
